# Human inherited complete STAT2 deficiency underlies inflammatory viral diseases

**DOI:** 10.1172/JCI168321

**Published:** 2023-06-15

**Authors:** Giorgia Bucciol, Leen Moens, Masato Ogishi, Darawan Rinchai, Daniela Matuozzo, Mana Momenilandi, Nacim Kerrouche, Catherine M. Cale, Elsa R. Treffeisen, Mohammad Al Salamah, Bandar K. Al-Saud, Alain Lachaux, Remi Duclaux-Loras, Marie Meignien, Aziz Bousfiha, Ibtihal Benhsaien, Anna Shcherbina, Anna Roppelt, COVID Human Genetic Effort, Florian Gothe, Nadhira Houhou-Fidouh, Scott J. Hackett, Lisa M. Bartnikas, Michelle C. Maciag, Mohammed F. Alosaimi, Janet Chou, Reem W. Mohammed, Bishara J. Freij, Emmanuelle Jouanguy, Shen-Ying Zhang, Stephanie Boisson-Dupuis, Vivien Béziat, Qian Zhang, Christopher J.A. Duncan, Sophie Hambleton, Jean-Laurent Casanova, Isabelle Meyts

**Affiliations:** 1 Laboratory of Inborn Errors of Immunity, Department of Microbiology, Immunology and Transplantation, KU Leuven, Leuven, Belgium.; 2 Department of Pediatrics, Leuven University Hospitals, Leuven, Belgium.; 3 St. Giles Laboratory of Human Genetics of Infectious Diseases, Rockefeller Branch, The Rockefeller University, New York, New York, USA.; 4 Laboratory of Human Genetics of Infectious Diseases, Necker Branch, INSERM U1163, Necker Hospital for Sick Children, Paris, France.; 5 University of Paris Cité, Imagine Institute, Paris, France.; 6 Department of Immunology, Great Ormond Street Hospital, London, United Kingdom.; 7 Division of Immunology, Boston Children’s Hospital and Harvard Medical School, Boston, Massachusetts, USA.; 8 King Abdullah Specialist Children’s Hospital and International Medical Research Center (KAIMRC), Riyadh, Saudi Arabia.; 9 College of Medicine, King Saud bin Abdulaziz University for Health Sciences, Riyadh, Saudi Arabia.; 10 Ministry of the National Guard–Health Affairs, Riyadh, Saudi Arabia.; 11 Pediatric Department, Section of Immunology and Allergy, King Faisal Specialist Hospital and Research Centre, Riyadh, Saudi Arabia.; 12 Gastroenterology, Hepatology and Nutrition Unit, University and Pediatric Hospital of Lyon, and Centre International de Recherche en Infectiologie (CIRI), INSERM U1111, Autophagy, Infection and Immunity, Lyon, France.; 13 Internal Medicine and Vascular Pathology Service, University Hospital of Lyon, Lyon, France.; 14 Clinical Immunology, Inflammation and Allergy Laboratory (LICIA), Faculty of Medicine and Pharmacy, King Hassan II University, Casablanca, Morocco.; 15 Clinical Immunology Unit, Pediatric Infectious Disease Department Children’s Hospital, Ibn Rochd University Hospital, Casablanca, Morocco.; 16 Department of Immunology, Dmitry Rogachev National Medical Research Center of Pediatric Hematology, Oncology and Immunology, Moscow, Russia.; 17 The COVID Human Genetic Effort is detailed in Supplemental Acknowledgments.; 18 Translational and Clinical Research Institute, Immunity and Inflammation Theme, Newcastle University, Newcastle upon Tyne, United Kingdom.; 19 Department of Pediatrics, Dr. von Hauner Children’s Hospital, University Hospital, Ludwig-Maximilians-University of Munich, Munich, Germany.; 20 Department of Virology, INSERM, Infection, Antimicrobiens, Modélisation, Evolution, UMR 1137, Bichat–Claude Bernard Hospital, University of Paris, Assistance Publique–Hôpitaux de Paris, Paris, France.; 21 Department of Paediatrics, Birmingham Chest Clinic and Heartlands Hospital, University Hospitals Birmingham NHS Foundation Trust, Birmingham, United Kingdom.; 22 Immunology Research Laboratory, Department of Pediatrics, King Saud University, Riyadh, Saudi Arabia.; 23 Pediatric Infectious Diseases Section, Beaumont Children’s Hospital, Royal Oak, Michigan, USA.; 24 Oakland University William Beaumont School of Medicine, Rochester, Michigan, USA.; 25 Department of Infectious Disease and Tropical Medicine, The Newcastle Upon Tyne Hospitals NHS Foundation Trust, Newcastle upon Tyne, United Kingdom, and; 26 Great North Children’s Hospital, The Newcastle Upon Tyne Hospitals NHS Foundation Trust, Newcastle upon Tyne, United Kingdom.; 27 Department of Pediatrics, Necker Hospital for Sick Children, Assistance Publique–Hôpitaux de Paris, Paris, France.; 28 Howard Hughes Medical Institute, New York, New York, USA.

**Keywords:** Immunology, Genetic diseases, Influenza, Innate immunity

## Abstract

STAT2 is a transcription factor activated by type I and III IFNs. We report 23 patients with loss-of-function variants causing autosomal recessive (AR) complete STAT2 deficiency. Both cells transfected with mutant *STAT2* alleles and the patients’ cells displayed impaired expression of IFN-stimulated genes and impaired control of in vitro viral infections. Clinical manifestations from early childhood onward included severe adverse reaction to live attenuated viral vaccines (LAV) and severe viral infections, particularly critical influenza pneumonia, critical COVID-19 pneumonia, and herpes simplex virus type 1 (HSV-1) encephalitis. The patients displayed various types of hyperinflammation, often triggered by viral infection or after LAV administration, which probably attested to unresolved viral infection in the absence of STAT2-dependent types I and III IFN immunity. Transcriptomic analysis revealed that circulating monocytes, neutrophils, and CD8^+^ memory T cells contributed to this inflammation. Several patients died from viral infection or heart failure during a febrile illness with no identified etiology. Notably, the highest mortality occurred during early childhood. These findings show that AR complete STAT2 deficiency underlay severe viral diseases and substantially impacts survival.

## Graphical abstract

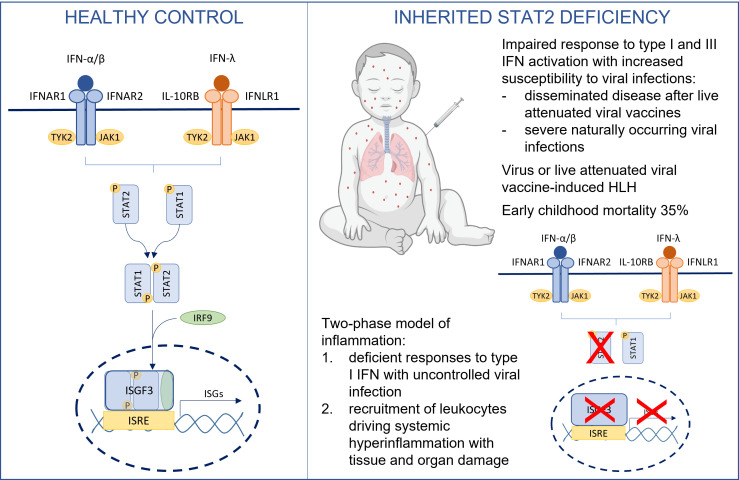

## Introduction

Human type I and III IFNs operate in almost all cell types (1, 2). Type I IFNs (IFN-α/β) are thought to be secreted by most, if not all, of the more than 400 discernable cell types of the human body (1). The secretion of type III IFNs (IFN-λ1–4) seems to be restricted to epithelial cells of barrier organs and plasmacytoid and conventional dendritic cells (3). The main signaling pathway downstream from the type I (IFNAR) and type III IFN receptors (IFNLR) involves STAT1 and STAT2 (4, 5). Once activated, these proteins combine with IFN regulatory factor 9 (IRF9) to form the IFN-stimulated gene factor 3 (ISGF3) complex (5). ISGF3 then translocates to the nucleus, where it binds IFN-stimulated response elements (ISREs) in the promoters of IFN-stimulated genes (ISGs) (5). Signaling via both receptors also induces the formation of STAT1 homodimers, which induce different ISGs through the binding of γ-activated sequence (GAS) elements in their promoters (5). STAT2 is also crucial for downregulating type I IFN responses by the recruitment of ubiquitin-specific protease USP18 to IFNAR2 (6). *STAT2* variants preventing interaction with USP18 and autosomal recessive USP18 deficiency result in a type I interferonopathy with uncontrolled inflammation (7–9).

Autosomal recessive (AR) complete STAT1 deficiency, reported in 24 patients from 15 kindreds (10, 11), was described in 2003 as underlying severe viral infections due to impaired type I and III IFN signaling and of mycobacterial disease due to impaired type II IFN signaling (10, 12, 13). In these patients, clinical outcome is poor without hematopoietic stem cell transplantation (HSCT), with death invariably occurring before the age of 3 years (10–12). AR STAT2 deficiency has been identified in 11 patients from 5 kindreds (14–18). These patients suffered disseminated viral disease, encephalitis, or systemic inflammation after vaccination with live attenuated viral vaccine (LAV) measles-mumps-rubella (MMR), and severe influenza pneumonia was reported in 1 case (14–18). Another STAT2-deficient child with a similar phenotype was recently reported with critical COVID-19 pneumonia (19).

Patients with inherited complete IFNAR2 (20, 21), IRF9 (22, 23), and IFNAR1 (24–26) deficiency have also been described. AR IFNAR2 deficiency was found in 5 patients from 4 families who presented with lethal encephalitis or hemophagocytic lymphohistiocytosis (HLH) following exposure to live attenuated MMR vaccine or with viscerotropic disease after live attenuated yellow fever virus (YFV) vaccination (20, 21, 27, 28). AR IRF9 deficiency has been reported in 3 children from 2 kindreds suffering from influenza pneumonia, recurrent infections, and disseminated disease after inoculation with LAVs (MMR and varicella zoster virus [VZV]) (22, 23). Finally, AR IFNAR1 deficiency underlies susceptibility to invasive disease after LAV inoculation (MMR and YFV), as described in 6 children from 4 families (24–26). In addition, 1 child suffering from herpes simplex virus type 1 (HSV-1) encephalitis, 3 children with critical COVID-19 pneumonia, and 2 previously well adult patients suffering from critical COVID-19 were recently found to harbor biallelic loss-of-function *IFNAR1* variants (19, 26, 29–31). Moreover, 2 recent studies revealed common (frequency >1%) null alleles of *IFNAR1* and of *IFNAR2* in Polynesians and Inuits, respectively (32, 33). Seven children with IFNAR1 deficiency and 5 with IFNAR2 deficiency displayed severe disease after LAVs and natural viral infections (32–34). Together, these 5 inborn errors underlie life-threatening infection following vaccination with LAV or severe influenza, SARS-CoV-2, enterovirus, and HSV-1 infections, highlighting the importance of type I IFNs for human antiviral immunity (10–12, 14, 20, 22–24). In this study, we analyzed the genetic, immunological, and clinical features of 23 patients with AR STAT2 deficiency from 10 kindreds and 7 countries, 11 of whom have been described elsewhere (14–18).

## Results

### Biallelic STAT2 variants in 10 kindreds.

**Figure 1:**
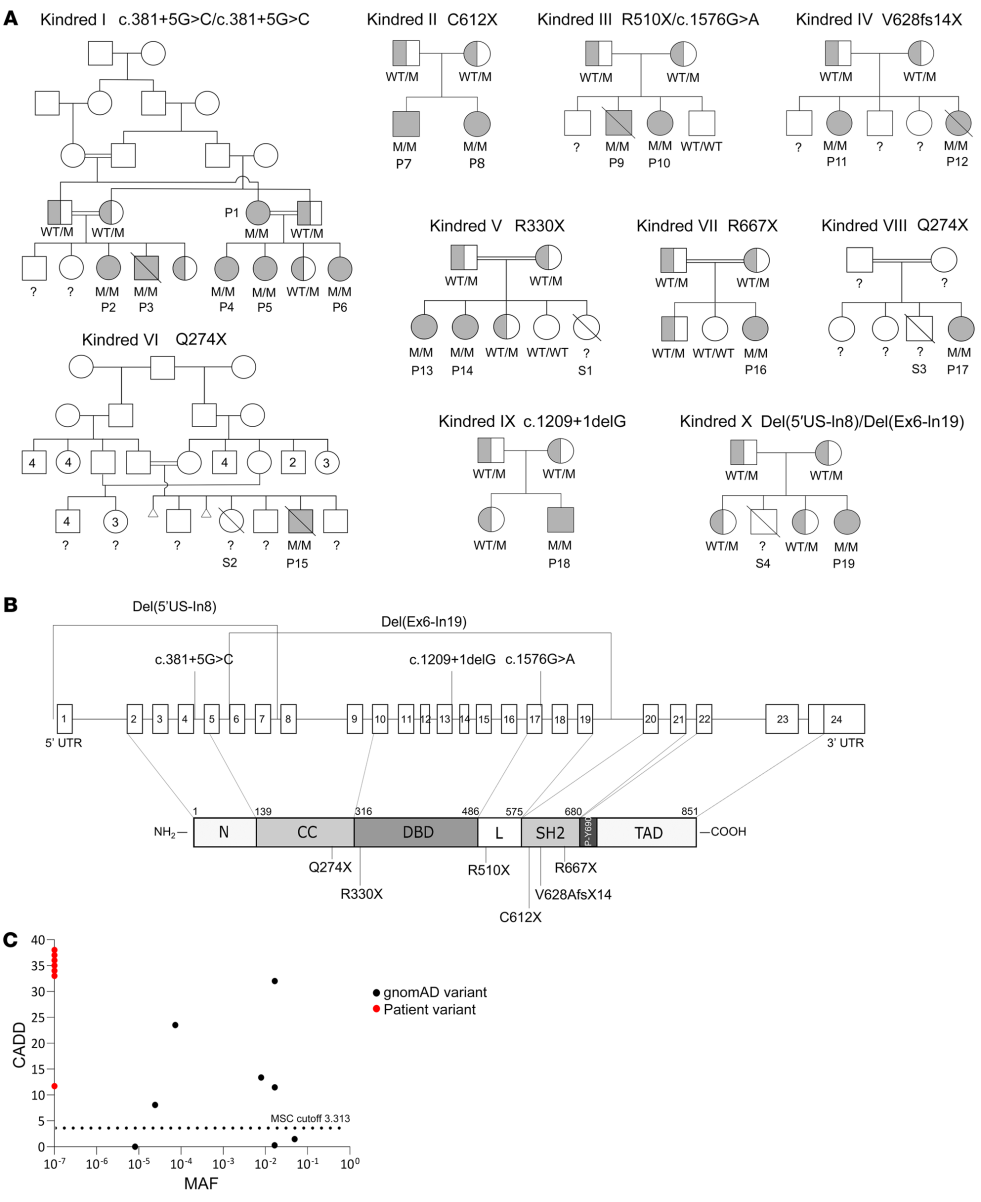
*STAT2* variants in 10 kindreds with severe viral infections. (**A**) Pedigrees of the 10 STAT2-deficient kindreds. Double lines connecting parents indicate consanguinity. Filled symbols indicate individuals with biallelic mutations, and half-filled symbols indicate carriers of heterozygous mutations. M, mutated allele; ?, unknown genotype. (**B**) Schematic illustration of the *STAT2* gene with 22 coding exons and of the STAT2 protein with its domains. N, N-terminal domain; CC, coiled-coil domain; DBD, DNA-binding domain; L, linker domain; SH2, Scr homology 2 domain; P-Y690, tyrosine phosphorylation site; TAD, transcriptional activation domain. All previously reported and new STAT2 variants are indicated. (**C**) Population genetics of homozygous coding missense and pLOF *STAT2* mutations from gnomAD and in-house cohorts. The patients’ variants are private and shown in red, whereas the 8 variants detected in gnomAD are shown in black. CADD, combined annotation-dependent depletion; MSC, mutation significance cutoff.

We studied 19 patients from 10 kindreds carrying biallelic *STAT2* variants and 4 deceased siblings for whom no genetic material was available. Two of the kindreds were from the United Kingdom (kindred I, patient 1–patient 6 [P1–P6], originally from Pakistan, described by Hambleton et al., ref. 14, and kindred II, P7 and P8, originally from Albania, described by Shahni et al., ref. 15), 1 was from Belgium (kindred III, P9 and P10, described by Moens et al., ref. 16), 2 were from North Africa (kindred IV, P11 and P12, Algerian, living in France, and kindred V, P13 and P14 and sibling 1, Moroccan), 2 were from Saudi Arabia (kindred VI, P15 and sibling 2, and kindred VIII, P17 and sibling 3), 1 was from Honduras (kindred VII, P16, living in the USA, described by Freij et al., ref. 17), 1 was from Nepal (kindred IX, P18, living in the USA, described by Alosaimi et al., ref. 18), and 1 was from Russia (kindred X, P19, and sibling 4) (Figure 1A). All cases were familial, and 5 kindreds were independently consanguineous (kindreds I, V, VI, VII, and VIII), according to family history. We identified 7 single-nucleotide substitutions, occurring in all domains of *STAT2* (c.381+5G>C; c.1576G>A; c.1528C>T [R510X]; c.1836C>A [C612X]; c.988C>T [R330X]; c.820C>T [Q274X]; c.1999C>T [R667X]), 2 small deletions (c.1883_1884del [V628fs14X]; c.1209+1delG), and 2 large deletions (del 8687 bp 5′ upstream–intron 8 [chr12:56360796-56352109]; del 7422 bp exon 5–intron 19 [chr12:56355504-56348082, human genome version GRCh38]), including the 6 previously reported variants (14–18) (Figure 1B and Table 1). All the *STAT2* variants were private, absent from public databases (gnomAD, version v2.1, https://gnomad.broadinstitute.org/; 1000 Genomes, http://www.internationalgenome.org), and predicted to be loss-of-function (pLOF) (Table 1). The segregation of these variants conformed to an AR model of inheritance. In 8 kindreds, the patients were homozygous for 1 of 7 variants, whereas in 2 kindreds, the patients were compound heterozygous (kindreds III and X) (Figure 1A and Table 1). Only 8 nonsynonymous variants have been reported in the homozygous state in the general population; these were missense variants with minor allele frequencies (MAF) between 10^–5^ and 0.05 (Figure 1C). Consistently, *STAT2* is subject to negative selection, as shown by its consensus negative selection (CoNeS) score of –1.21 (35).

### Severe disease following LAV vaccination.

Seventeen patients received LAVs: 12 patients received MMR vaccine (P2, P4, P7, P8, P9, P10, P11, P12, P13, P14, P15, sibling 1), 1 patient measles vaccine (P17), 1 patient both the measles and MMR vaccines (sibling 3), and 3 patients both the MMR and VZV vaccines (P16, P18, sibling 2). All patients were vaccinated between the ages of 9 months and 2 years. Five patients were not vaccinated with LAV (P3, P5, P6, P19, sibling 4), and for P1, information was unavailable. Clinical disease developed in 12 of the 17 vaccinated patients (information unavailable for 3 patients): measles with systemic inflammation, atypical Kawasaki disease and encephalitis or meningitis in 2 cases (P7, P11); measles with systemic inflammation and multiple organ involvement (pneumonia, hepatitis, coagulopathy) in 4 cases (P2, P8, P9, P10); prolonged febrile sickness with or without a rash in 3 cases (P4, P17, sibling 3), 1 of whom suffered sensorineural hearing loss; mumps in 2 cases (P15, sibling 3), accompanied by sensorineural hearing loss, systemic inflammation and atypical Kawasaki disease in P15; respiratory insufficiency and meningitis in 1 case (P18); and chickenpox in 1 case (P16). P16 and P18 also presented secondary HLH a few days after inoculation with MMR and VZV vaccines. Human herpesvirus 6 (HHV6) viremia was detected in these patients at the time. PCR for measles, mumps, and rubella was not performed in P16 (although VZV PCR was positive on a cutaneous lesion), but the vaccine-strain mumps virus was detected in cerebrospinal fluid from P18. Diagnosis in the other patients was based on clinical presentation and timing relative to LAV vaccination; it was also confirmed by PCR in P8 (PCR on throat swab positive for measles, mumps, and rubella and PCR on cerebrospinal fluid positive for mumps), P11 (PCR on throat swab positive for mumps and rubella), and P15 (PCR on urine positive for mumps and IgM against mumps virus detected in serological tests).

### Other severe viral infections.

Ten of the 23 patients suffered from severe naturally occurring viral illnesses (Table 2 and Table 3). Six unrelated patients had PCR-proven influenza A pneumonia (P2, P10, P12, P15, P16, sibling 2), 2 with acute respiratory distress syndrome (ARDS) requiring mechanical ventilation (P15, P16). One patient had mild influenza A that was treated with oseltamivir and resolved uneventfully (P18). Two patients had enteroviral meningitis (P10: coxsackievirus B, P11), 1 patient had enteroviral colitis with exudative enteropathy (P12), and 1 patient had hepatitis and pneumonia during infection with adenovirus, enterovirus, and respiratory syncytial virus (RSV) (P9). Herpesvirus infections were not systematically severe in this cohort, although 2 patients experienced HLH with cytopenia concomitant with HHV6 infection or reactivation shortly after MMR vaccination (P16 and P18), and 1 patient (sibling 1) died from encephalitis due to primary HSV-1 infection, initially manifesting as gingivostomatitis. Three patients had stomatitis due to HSV-1 infection (P2, P15, sibling 4), with concomitant herpetic keratitis in 1 (P15). Four patients suffered from EBV infection (P2, P10, P17, P19), 3 with a mild or asymptomatic course. P10 had fever, lymphadenopathy, and splenomegaly, with persistent virus detected in the blood and cerebrospinal fluid over a period of 3 years.

One 19-year-old patient (P11) developed severe COVID-19 pneumonia with involvement of 75% of the lungs, requiring noninvasive ventilation. She had not been vaccinated against SARS-CoV-2. She was treated with steroids, antibiotics, and the anti–SARS-CoV-2 monoclonal antibodies casirivimab and imdevimab and recovered. P10 had mild COVID-19 at the age of 17 years and was treated with the anti–SARS-CoV-2 monoclonal antibody sotrovimab on day 4 (D4) after symptom onset; she had previously received 3 doses of anti–SARS-CoV-2 vaccine (Comirnaty). Viremia was undetectable on D4, prior to administration of sotrovimab, and the patient recovered without complications. P1, P2, and P16 also had mild COVID-19 manifesting as a febrile upper respiratory tract infection at the age of 40, 14, and 3 years, respectively. P1 had previously received 2 doses of anti–SARS-CoV-2 vaccine, while P2 and P16 had not been vaccinated, and they all recovered uneventfully without treatment. P19, also unvaccinated, had asymptomatic COVID-19 at 12 years of age (infection proved by a positive PCR on a nasopharyngeal swab) and developed T cell immunity to the virus, as demonstrated by ELISpot (data not shown).

### Benign viral infections.

Fifteen of the 23 patients suffered from various mild or asymptomatic viral infections. One patient had IgG against CMV without clinical illness (P18). Three patients had uneventful natural chickenpox (P4, P5, P14), whereas 1 patient had a more severe form (skin and mucosae) accompanied by recurrent common warts and molluscum contagiosum (P10). Finally, 10 patients experienced recurrent respiratory tract or gastroenteric infections with several viruses, in some cases requiring hospitalization for supportive therapy (P4, P5, P6, P9, P10, P12, P13, P16, P18, P19). The viruses involved (infections proved by PCR) included rhinovirus/enterovirus, rotavirus, adenovirus, norovirus, RSV, parainfluenza virus, coronavirus HKU1, and human metapneumovirus. Bacterial infections were clinically diagnosed in 13 patients. Eight of these patients had a clinical diagnosis of pneumonia, 1 with isolation of *Pseudomonas aeruginosa* and *Stenotrophomonas* from bronchoalveolar lavage (P15) and 7 without microbial isolation (P9, P10, P13, P14, P17, P19, sibling 1). No mycobacterial, fungal, or parasitic infections were reported. Nine patients were vaccinated with live bacillus Calmette-Guérin (BCG) without complications. VirScan analysis for P10 and P12 is reported in Supplemental Table 1 (supplemental material available online with this article; https://doi.org/10.1172/JCI168321DS1) (36).

### Inflammatory manifestations.

Systemic inflammation, manifesting as an increase in serum proinflammatory cytokine concentrations and multiple organ dysfunction, or (atypical) Kawasaki disease, was diagnosed or suspected in 6 patients (after LAV vaccination in 5), and inflammation and cytopenia compatible with secondary HLH were present in 2 patients. P7 had a first episode of meningoencephalitis and opsoclonus-myoclonus 1 month after vaccination with MMR and sustained substantial neurological damage following a second episode of meningoencephalitis, with seizures and opsoclonus-myoclonus triggered by an undetermined infection with fever and diarrhea at the age of 2.5 years. P11 was diagnosed with atypical Kawasaki disease after MMR vaccination. Five patients (P8, P9, P10, P16, P18) had transient neutropenia, anemia, and thrombocytopenia, with or without coagulopathy, coinciding with systemic inflammation and multiple organ involvement during febrile illness, LAV administration, or viral infections, indicative of secondary HLH, although diagnosis based on HLH criteria was confirmed in only 2 patients (P16, P18). The determination of a panel of proinflammatory cytokines in serum samples from P10 outside clinical events (*n* = 2) and during mild COVID-19 (*n* = 1, 4 days from symptom onset) showed an increase in IL-6 levels during infection (Supplemental Figure 1A).

### Immunological phenotype.

Five of 12 patients (P8, P9, P10, P11, P17) had T and/or B cell lymphopenia during acute illness, with documented normalization upon resolution of the illness in 4 cases (data not shown). We also performed in-depth immunophenotyping by mass cytometry on PBMCs derived from P10 outside of infectious episodes at the age of 17 years. No notable differences with respect to a healthy control were detected (Supplemental Figure 1B). Mitogen-induced T cell proliferation was normal in the 5 patients tested. Total IgG and IgM levels were normal or high in all 11 patients tested (during Ig supplementation in 4 cases). Partial IgA deficiency was detected in 4 of the 11 patients tested, all under the age of 4 years, which is in line with findings in the general population of this age. Specific postvaccination antibody levels were normal in all 7 patients tested for tetanus and in both patients tested for pneumococcus. P10 mounted normal responses to the mRNA vaccine against SARS-CoV-2 (Supplemental Table 2).

### Outcome and treatment.

**Figure 2:**
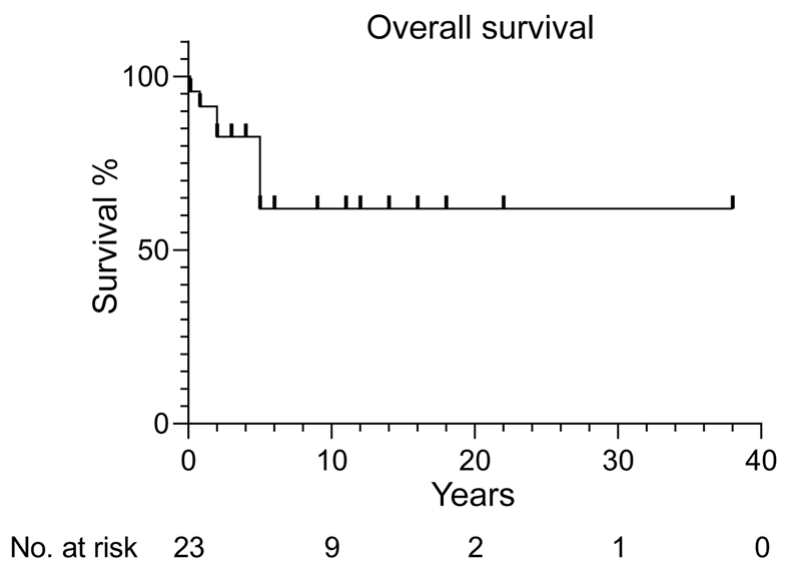
Overall survival of 23 patients with STAT2 deficiency. Ticks on the graph represent censored data.

Eight patients died in early childhood (range: 2 months to 7 years). Six patients died from heart failure in the context of a febrile illness without identified viral or other infectious etiology (P3, P9, P12, P15, sibling 2, sibling 3). In 2 cases, the clinical diagnosis before death was tonsillitis (P12, P15), but no microbiological samples were obtained. One 5-year-old child died from fulminant hepatitis of possible but unproven viral etiology (sibling 4), and one 2-year-old child died from HSE (sibling 1). In P3, postmortem examination revealed enterocolitis, interstitial pneumonia, and cerebral cortical edema with neuronal apoptosis, consistent with overwhelming viral illness, and in sibling 4, the results were consistent with overwhelming infection. One patient was on cotrimoxazole prophylaxis at the time of death, whereas the other patients were not on any medication (in P3, P9, P15, and all siblings, death preceded the genetic diagnosis). Three patients (P7, P10, P18) received high-dose intravenous immunoglobulins (0.8 g/kg to 2 g/kg) during systemic inflammation or Kawasaki disease, resulting in a rapid resolution of symptoms. Several patients were treated empirically with antibiotics, antiviral drugs (acyclovir), and antifungal agents during febrile illnesses with multiple organ involvement, cytopenia, and/or coagulopathy, although no bacterial or fungal infections were proven, and 2 patients received corticosteroids (P7, P10). Fifteen patients are still alive at this writing (median age: 13 years; range: 5–40 years). Four of these patients are on acyclovir prophylaxis (aged 8–20 years), and 6 are on intravenous or subcutaneous immunoglobulins (aged 5–13 years), combined with cotrimoxazole prophylaxis in 2 patients. None of these patients has undergone HSCT. Overall survival was assessed (Figure 2), and mortality was found to be 35% in early childhood.

### Expression and function of STAT2 mutant alleles.

**Figure 3:**
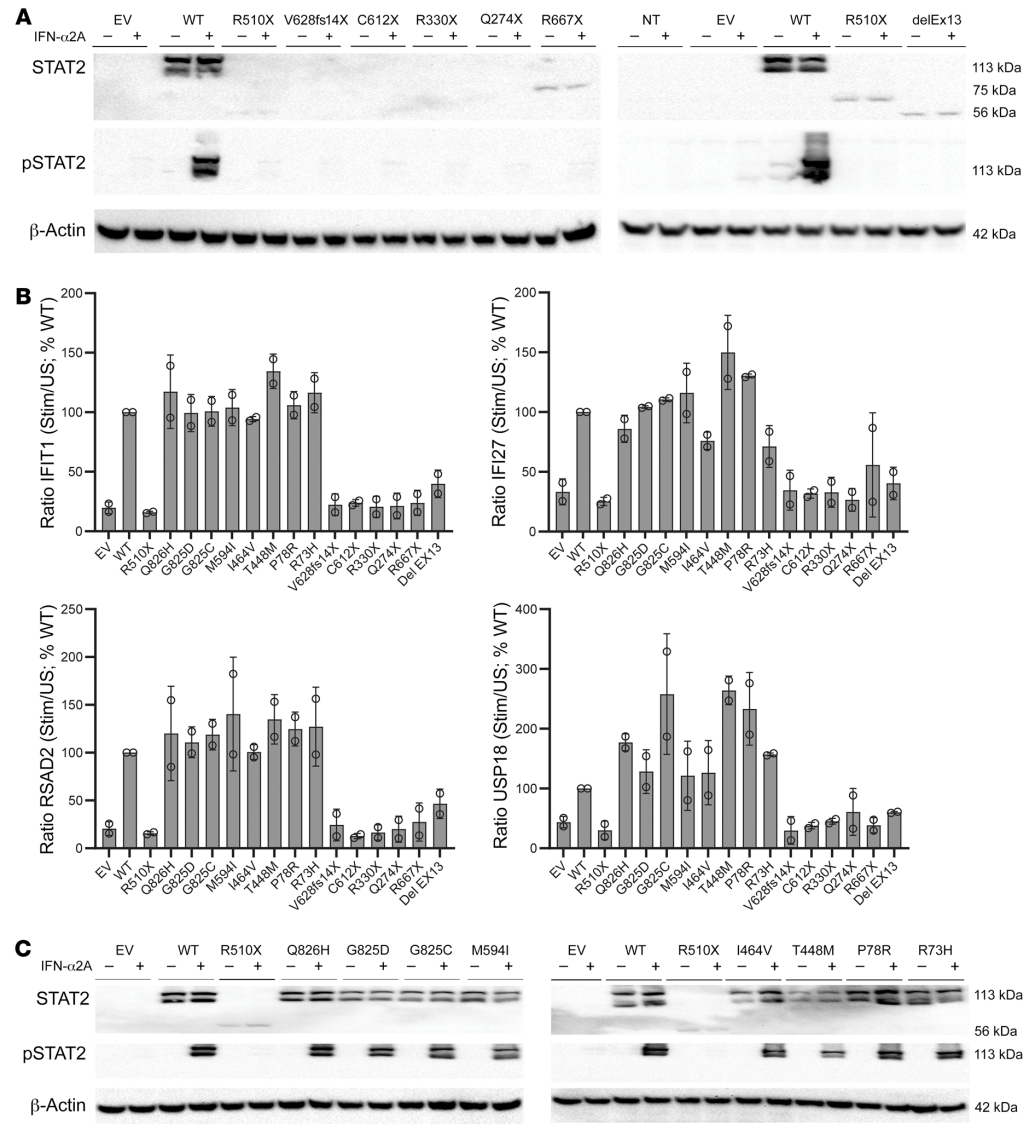
Impact of *STAT2* variants on protein production and type I IFN signaling. (**A**) Immunoblot of STAT2 and phosphorylated STAT2 in HEK293T cells transfected with an untagged pCMV6 expression vector containing either the WT STAT2 cDNA or 1 of the variant cDNAs in basal conditions (–) or after pretreatment with 10,000 U/mL IFN-α2A for 30 minutes (+). One representative blot from 3 experiments performed is shown. NT, not transduced; EV, empty vector. (**B**) Transcription levels for *IFIT1*, *IFI27*, *RSAD2*, and *USP18* assessed by RT-qPCR on U6A fibrosarcoma cells transfected with empty vector, WT STAT2, one of the mutated alleles, or one of the homozygous variants found in gnomAD after pretreatment with 10,000 U/mL of IFN-α2B for 6 hours. The mean (*n* = 3) and SEM are shown. Results are normalized relative to WT unstimulated conditions. (**C**) Immunoblot of STAT2 and phosphorylated STAT2 in HEK293T cells transfected with an untagged pCMV6 expression vector containing either the WT STAT2 or one of the homozygous variants found in gnomAD in basal conditions (–) or after pretreatment with 10,000 U/mL IFN-α2A for 30 minutes (+). A representative blot from 2 experiments performed is shown.

We studied the effect of the *STAT2* alleles on STAT2 protein production and phosphorylation by transiently transfecting HEK293T cells with an untagged pCMV6 expression vector containing either WT *STAT2* or 1 of the variant alleles (c.1528C>T [R510X], c.1836C>A [C612X], c.1883_1884del [V628fs14X], c.988C>T [R330X], c.820C>T [Q274X], c.1999C>T [R667X], or delEx13 due to c.1209+1delG). The effect of the large deletions, del(5′US-In8)/del(Ex5-In19), was studied in an EBV-transformed lymphoblastoid cell line (EBV-LCL) obtained from P19. Immunoblotting demonstrated a complete absence of the STAT2 protein (C612X, v628fs14X, R330X, Q274X, del[5′US-In8]/del[Ex5-In19]) or a truncated protein (R667X, R510X, delEx13) in this overexpression system and an absence of phosphorylation of the Y-690 residue of STAT2 after stimulation with IFN-α2A for all alleles (Figure 3A). We then introduced the STAT2 alleles into STAT2-deficient fibrosarcoma cells and performed quantitative reverse-transcriptase PCR (RT-qPCR) to evaluate upregulation of the ISGs *IFIT1*, *IFI27*, *RSAD2*, and *USP18* after stimulation with IFN-α2A. We evaluated the upregulation of *MX1*, *RSAD2*, and *USP18* in LCLs from P19. ISG induction was suppressed by all variant alleles, but was normal for the WT allele (Figure 3B). We also tested the missense variants found in the homozygous state in the general population for their expression and phosphorylation of STAT2 protein and for the upregulation of ISGs after IFN-α2A treatment. These were normal relative to WT STAT2 (Figure 3, B and C). We concluded that the patients’ alleles resulted in a loss of expression of full-length protein and a loss of function of STAT2, at least in this experimental setting.

### Single-cell RNA-Seq reveals impaired basal ISG expression in STAT2-deficient leukocytes.

**Figure 4:**
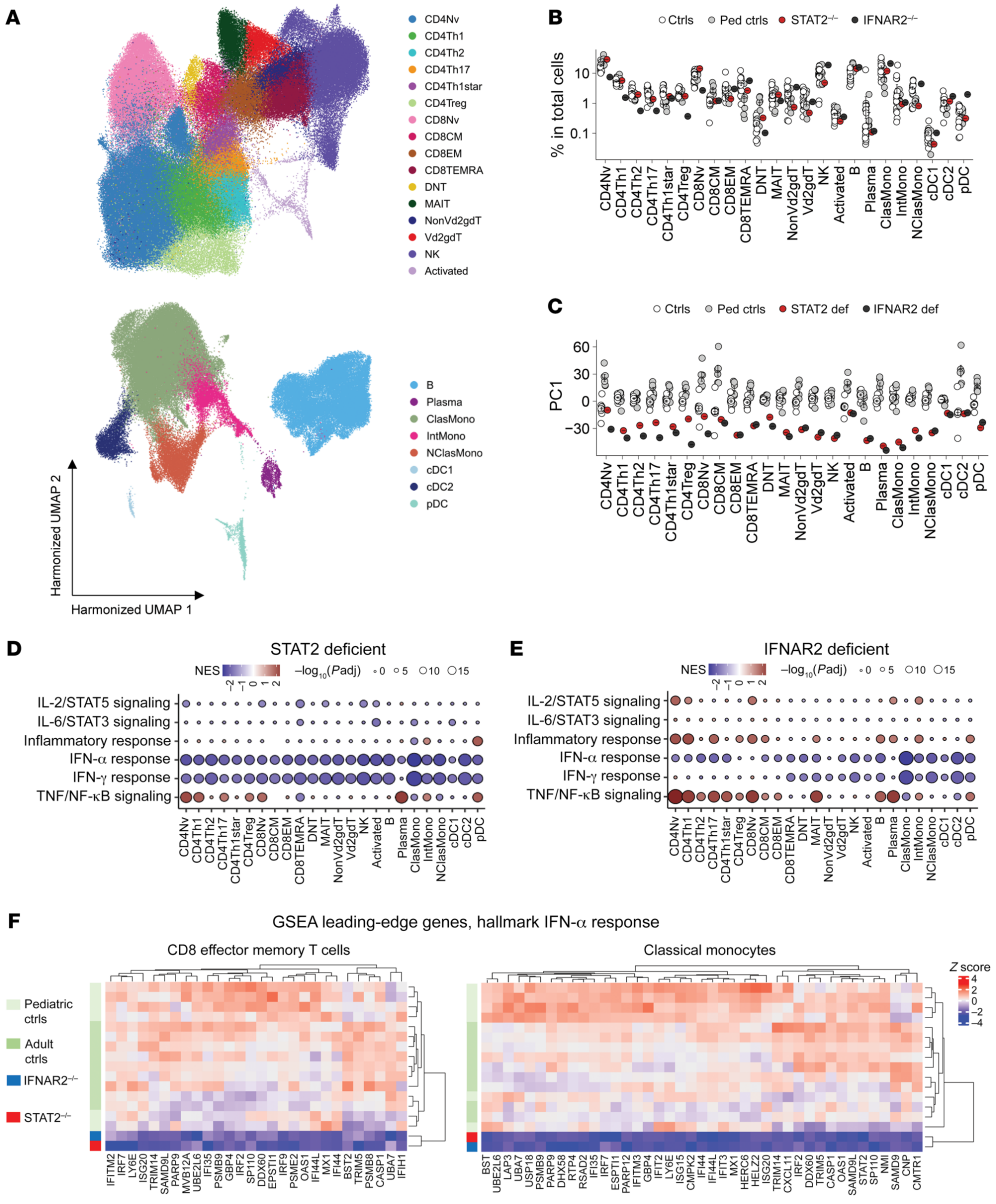
STAT2 deficiency impairs cellular responses to type I IFN in the basal state without affecting leukocyte subsets. scRNA-Seq was performed on PBMCs from a STAT2-deficient patient (P10) and controls. (**A**) Leukocyte subsets identified by clustering analysis. (**B**) Relative abundance of cell types among PBMCs. (**C**) Pseudobulk PCA. (**D** and **E**) GSEA. Genes ranked based on fold change differences in expression in the STAT2-deficient patient (**D**) or the IFNAR2-deficient patient (**E**) relative to healthy pediatric controls were projected onto the hallmark gene sets (http://www.gsea-msigdb.org/gsea/msigdb/genesets.jsp?collection=H). Six immune-related gene sets were chosen for visualization. NES, normalized enrichment score. (**F**) Normalized pseudobulk read counts for GSEA leading-edge genes for the hallmark IFN-α response gene set common to the STAT2-deficient patient and pediatric controls and to the IFNAR2-deficient patient and pediatric controls, as shown in **D** and **E**. Representative results for CD8EM cells and classical monocytes are shown.

**Figure 5:**
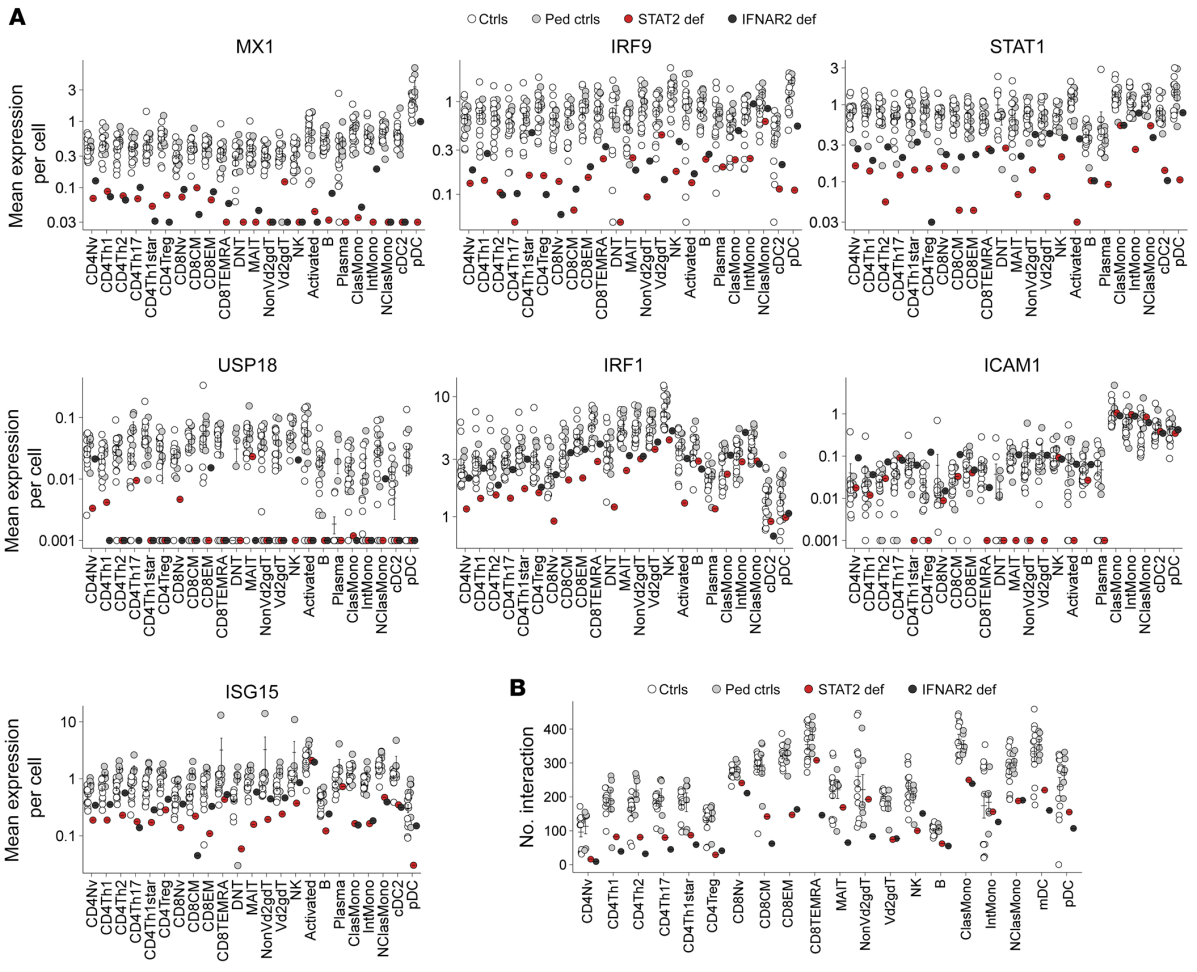
ISG expression and intercellular interactions between CD8EM cells and classical monocytes are weak in STAT2-deficient patients. scRNA-Seq was performed on PBMCs from a STAT2-deficient patient (P10) and controls. (**A**) Mean single-cell expression levels for representative IFN-stimulated genes (logarithmic scale). (**B**) CellChat analysis showing the crude number of predicted cell-cell interactions.

We then investigated the impact of STAT2 deficiency on leukocyte subsets, their transcriptomic profile, and their predicted molecular interactions by single-cell RNA sequencing (scRNA-Seq) on PBMCs from P10 and a patient with IFNAR2 deficiency in clinical remission compared with healthy pediatric controls (37). Clustering analysis identified 24 different leukocyte subsets, none of which was altered in the STAT2-deficient patient (Figure 4, A and B). Pseudobulk principal component analysis (PCA) and gene-set enrichment analysis (GSEA) (38, 39) revealed a distinctive transcriptional pattern across multiple leukocyte subsets that was common to STAT2- and IFNAR2-deficient patients (Figure 4C). ISGs, which are mostly induced by type I and/or type II IFNs, were downregulated in all 24 leukocyte subsets in the STAT2- and IFNAR2-deficient patients, with *MX1*, *IRF9*, *USP18*, and *ISG15* all downregulated in CD8^+^ effector memory T cells (CD8EM cells) and classical monocytes (Figure 4, D–F, and Figure 5, A and B). The single-cell expression of *MX1*, *IRF9*, *STAT1*, *IRF1*, *ICAM1*, *ISG15*, and *USP18* was also downregulated (Figure 4F). In contrast, a significant upregulation of genes involved in TNF/NF-κB signaling was observed in 8 leukocyte subsets in STAT2- and IFNAR2-deficient patients (Figure 4, D and E). Intercellular communication analysis with CellChat (40) indicated that intercellular interaction between CD8EM cells and classical monocytes was weaker in STAT2- and IFNAR2-deficient patients (Figure 5B and Supplemental Figure 2A). CD8^+^ central memory T cells (CD8CM cells) and CD8EM cells were the only cell types for which the classical monocytes of STAT2- and IFNAR2-deficient patients were predicted to provide weaker signals. In contrast, STAT2- and IFNAR2-deficient CD8EM cells were predicted to receive weaker signals from a wide range of cell types, with differential expression of the galectin- and MHC-I–related pathways (Supplemental Figure 2, A–D). These data confirm that STAT2 deficiency impairs cellular responses to type I IFN in the basal state without affecting leukocyte subsets per se. They also highlight the upregulation of the TNF/NF-κB signature.

### IFN-α fails to induce ISG expression in STAT2-deficient leukocytes.

**Figure 6:**
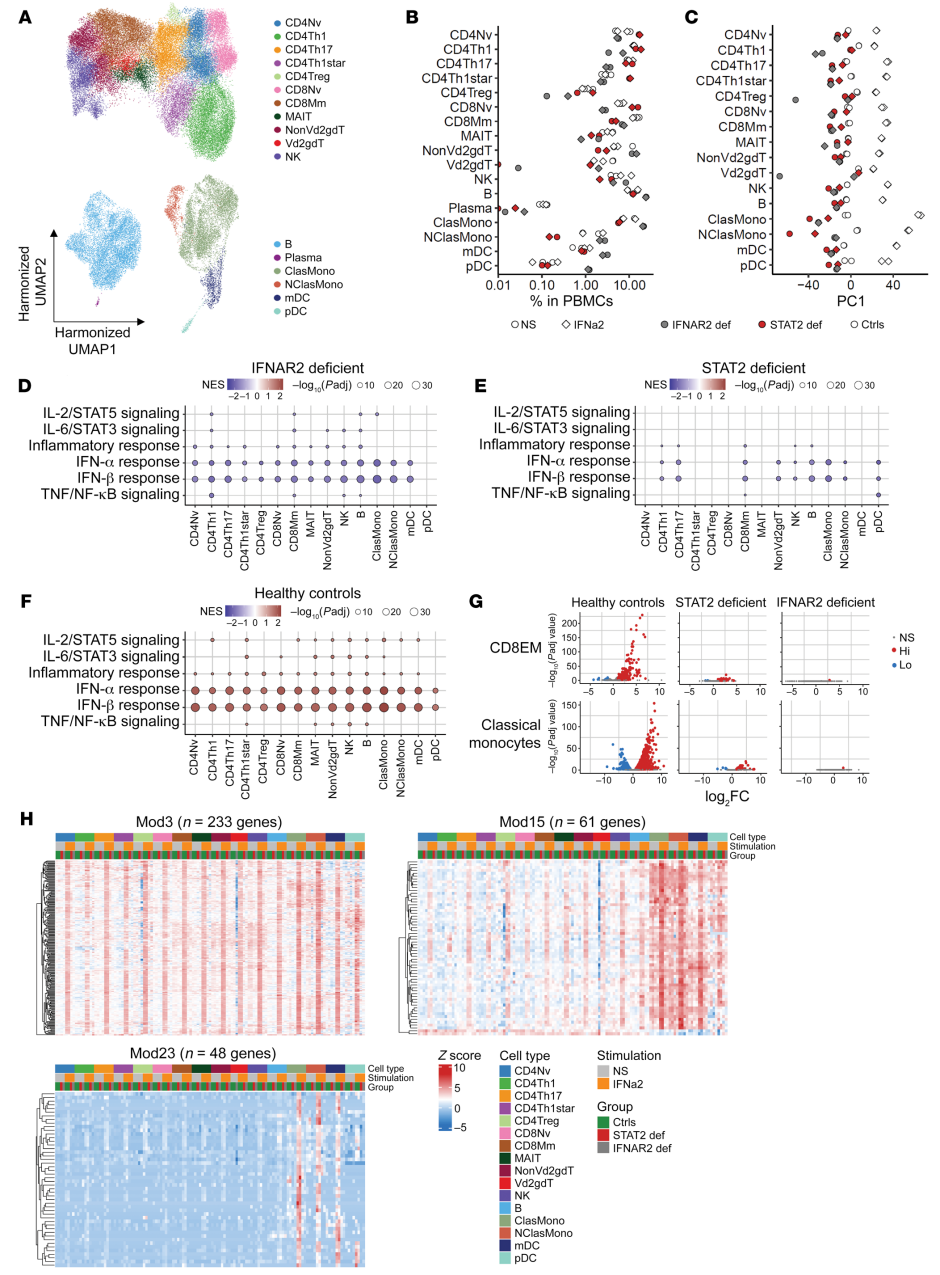
IFN-α fails to induce ISG expression in STAT2 deficient leukocytes. (**A**) Leukocyte subsets identified by clustering analysis. PBMCs were incubated for 6 hours with or without IFN-α2b (1000 IU/mL). Variations due to batch and stimulation were integrated with Harmony (see Supplemental Methods). (**B**) Relative abundance of the identified cell types among PBMCs after 6 hours of incubation without (circles) and with (squares) IFN-α2b. (**C**) Pseudobulk PCA for leukocyte subsets. (**D**–**F**) GSEA in IFN-α2b–stimulated PBMCs from healthy controls and STAT2- and IFNAR2-deficient patients. Ranking of gene differential expression projected onto the hallmark gene sets. Immune-related gene sets were chosen for visualization. (**D**) IFNα2b-treated versus nonstimulated for healthy controls.(**E**) Relative fold-change difference in expression in the STAT2-deficient patient relative to controls for the expression changes induced by IFNα2b stimulation versus nonstimulation. (**F**) Relative fold-change difference in expression in the IFNAR2-deficient patient relative to controls for differences in expression induced by IFNα2b stimulation versus nonstimulation. (**G**) Representative volcano plots. Genes significantly up- and downregulated (FDR-adjusted *P* value < 0.05 and |log_2_FC| > 1) are shown in red and blue, respectively. (**H**) WGCNA. Three modules of coexpressed genes (Mod3/15/23) were induced by IFN-α2b in control cells. These modules were used for the heatmap analysis. Heatmaps show batch-corrected *Z*-transformed normalized pseudobulk read counts.

We investigated STAT2-dependent cellular responses to IFN-α by performing scRNA-Seq in unstimulated or IFN-α2B–stimulated PBMCs from healthy controls, a STAT2-deficient patient, and an IFNAR2-deficient patient. Stimulation with IFN-α2B did not affect the distribution of the 17 leukocyte subsets identified in clustering analysis (Figure 6, A and B). Pseudobulk PCA showed weaker transcriptional responses to IFN-α2B stimulation across all cell types analyzed in STAT2- and IFNAR2-deficient cells relative to healthy controls (Figure 6C). GSEA with the hallmark signature gene sets revealed a significant impairment of the induction of type I ISGs by IFN-α2B across multiple cell types in the STAT2- and IFNAR2-deficient patients (Figure 6, D–G). Unlike IFNAR2-deficient cells, STAT2-deficient cells displayed a minimal residual response to type I IFN, which was most pronounced in classical and nonclassical monocytes (Figure 6, D–G). Weighted gene coexpression network analysis (WGCNA) (41) identified 3 modules of genes induced by IFN-α2B in control cells, but not in STAT2- and IFNAR2-deficient cells (Figure 6H). Module 3, which included *USP18*, *MX1*, *ISG15*, *OAS1-2-3*, *IL15*, *IRF7*, and *IRF9*, was induced across all lymphoid and myeloid leukocyte subsets analyzed, whereas modules 15 and 23, containing *JAK2*, *TRIM25-38-69*, *CXCL9-10-11*, and *UNC93B1*, were more prominently induced in myeloid cells. Overall, STAT2 deficiency was found to impair cellular responses to type I IFNs profoundly across leukocytes (Figure 6H).

### Impaired antiviral immunity to HSV-1 in STAT2-deficient SV40 fibroblasts.

We report the first case, to our knowledge, of HSV-1 encephalitis (HSE) in STAT2 deficiency (sibling 1). We infected SV40 fibroblasts from healthy controls and from STAT2-, IFNAR1-, STAT1-, and IRF9-deficient patients with HSV-1 in vitro. We observed at least a 10^7^-fold increase in HSV-1 titers relative to healthy cells in STAT2-deficient cells 48 hours after infection (Supplemental Figure 3A). Similar results were obtained for IFNAR1-, STAT1-, and IRF9-deficient cells. Pretreatment with IFN-α2B for 16 hours before HSV-1 infection significantly decreased viral replication levels relative to untreated cells 72 hours after infection in healthy control cells, but not in STAT2-, IFNAR1-, STAT1-, and IRF9-deficient cells (Supplemental Figure 3A). These data confirm the greater susceptibility to HSV-1 infection of nonhematopoietic STAT2-deficient cells in vitro.

### Lack of ubiquitin-specific peptidase 18 upregulation in patient-derived STAT2-deficient LCLs stimulated with IFN-α.

**Figure 7:**
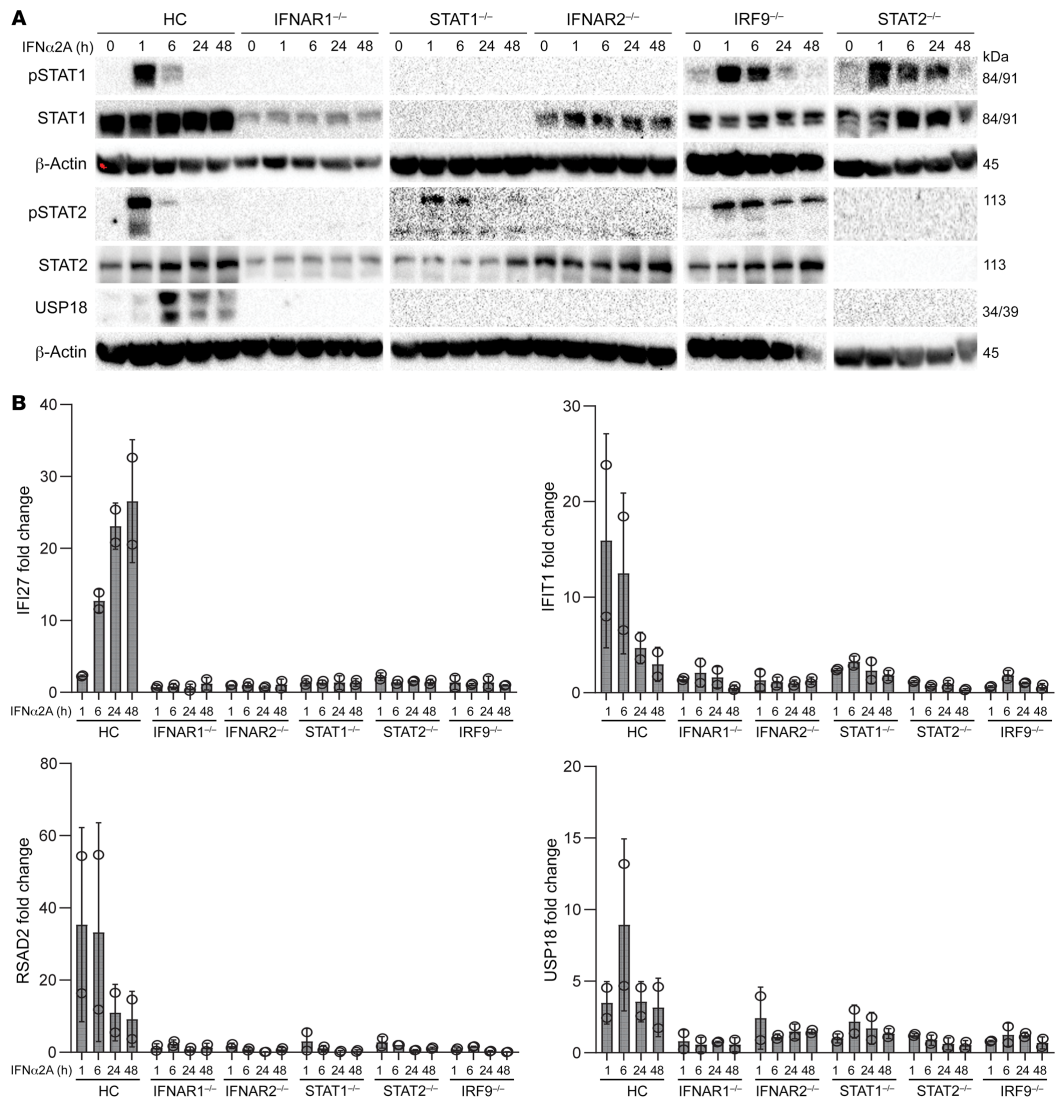
STAT2-deficient cells do not upregulate USP18. (**A**) Immunoblot of STAT1, phospho-STAT1, STAT2, phospho-STAT2, and USP18 in EBV-LCL cells derived from either a healthy control (HC) or a patient with complete IFNAR1, IFNAR2, STAT1, STAT2, or IRF9 deficiency after pretreatment with 10,000 U/mL IFN-α2A for 1, 6, 24, or 48 hours. One representative blot from 3 experiments performed is shown. (**B**) Transcription levels for *IFI27*, *IFIT1*, *RSAD2*, and *USP18* assessed by RT-qPCR on EBV-LCL cells derived from either a healthy control or a patient with complete IFNAR1, IFNAR2, STAT1, STAT2, or IRF9 deficiency after pretreatment with 10,000 U/mL IFN-α2A for 1, 6, 24 or 48 hours. The mean (*n* = 3) and SEM are shown. Results are normalized relative to unstimulated healthy control conditions.

Ubiquitin-specific peptidase 18 (USP18) is an ISG that is recruited to IFNAR2 by STAT2 and downregulates the response to type I IFNs through steric hindrance, preventing JAK1 from binding to IFNAR2 (6–8, 42, 43). Impaired USP18 upregulation may, therefore, theoretically contribute to the hyperinflammation observed in individuals with STAT2 deficiency, although hyperinflammation has been documented in patients with other inborn errors of type I IFN immunity (11, 20, 22, 26, 31–33). Nevertheless, we investigated the activation of the JAK/STAT pathway downstream from IFNAR in EBV-derived LCLs from a healthy control and patients with complete STAT2, STAT1, IRF9, IFNAR1, or IFNAR2 deficiency at various time points after stimulation with IFN-α2A. In the healthy control, USP18 expression was detected from 6 to 48 hours after IFN-α2A stimulation. However, no upregulation of USP18 protein levels was observed after IFN-α2A stimulation in STAT1-, STAT2-, IFNAR1-, IFNAR2-, or IRF9-deficient LCLs (Figure 7A and Supplemental Figure 3B). Moreover, phosphorylated STAT1 (phospho-STAT1) and phospho-STAT2 levels returned to baseline within 24 hours in healthy control LCLs, whereas they remained high for up to 24 hours after stimulation with IFN-α2A in STAT2- and IRF9-deficient LCLs. RT-qPCR showed a strong impairment of the induction of ISGs, including USP18, in STAT2-, STAT1-, IFNAR1-, IFNAR2-, and IRF9-deficient LCLs at all time points after stimulation with IFN-α2A (Figure 7B). These findings suggest that low levels of USP18 expression in response to IFN-α2A stimulation may contribute to the inflammatory phenotype in patients with STAT2 deficiency and in patients with STAT1 or IRF9 deficiency, but such mechanisms clearly cannot operate in patients with IFNAR1 or IFNAR2 deficiency. The phenotypic similarity of IFNAR1 and IFNAR2 deficiencies and STAT2 deficiency, in terms of inflammatory episodes, and the lack of type I IFN–induced ISG expression in STAT2-deficient LCLs suggest that the impaired induction of USP18 makes no major contribution to hyperinflammation in the absence of STAT2 (20, 32, 33).

### Prolonged STAT1 phosphorylation in patient-derived STAT2-deficient LCLs upon stimulation with IFN-α.

STAT1 homodimers can operate downstream from IFNAR in the absence of STAT2, potentially inducing the expression of genes harboring γ-activating sequences (GAS), thereby driving IFN-γ–like inflammatory responses (44). However, HLH also occurs in AR complete STAT1 deficiency (11). Nevertheless, we measured STAT1 production, STAT1 phosphorylation, and ISG induction in LCLs from a healthy control and patients with complete IFNAR1, IFNAR2, STAT1, STAT2 (P10 and P19), or IRF9 deficiency after stimulation with IFN-α2A for 1, 6, 24, or 48 hours. Phospho-STAT1 levels in STAT2- and IRF9-deficient LCLs and phospho-STAT2 levels in STAT1- and IRF9-deficient LCLs were similar to those of the control 1 hour after stimulation, but phosphorylation levels remained higher at the later time points than in the healthy control. We observed higher levels of baseline STAT1 phosphorylation in STAT2- and IRF9-deficient LCLs. STAT2-, STAT1-, IFNAR1-, IFNAR2-, and IRF9-deficient LCLs displayed a strong impairment of the induction of the ISRE-dependent ISGs *IFI27*, *IFIT1*, *RSAD2*, and *USP18* by RT-qPCR analyses 1, 6, 24, and 48 hours after stimulation with IFN-α2A (Figure 7B and Supplemental Figure 3C). We then assessed the upregulation of the classical IFN-γ–induced ISGs *IRF1* and *ICAM1*, which are GAS-dependent, in response to IFN-α2A and IFN-γ (Supplemental Figure 3, D and E). *IRF1* expression was induced in the healthy control and in IRF9-deficient patient cells, but was induced to a much lesser extent in STAT2-deficient patient cells following stimulation with IFN-α2A, whereas *ICAM1* expression was not upregulated. Thus, the prolonged STAT1 phosphorylation observed in STAT2-deficient LCLs after IFN-α2A stimulation was not accompanied by increased expression of *IRF1* and *ICAM1*, 2 typical IFN-γ–dependent ISGs, at least in these experimental conditions. We also found no significant difference in the expression of *SOCS1*, *SOCS3*, and *CIITA* between the tested cell lines at any time point after stimulation with IFN-α2A (data not shown).

### Enhanced inflammatory response in the basal state and during COVID-19 in individuals with STAT2 deficiency.

We next analyzed the differential gene expression in whole blood from a STAT2-deficient patient (P10) during acute mild COVID-19 due to PCR-confirmed natural SARS-CoV-2 infection (D4 after symptom onset) or in the basal state, 5 months after infection, relative to a healthy control matched with the patient for age and sex. GSEA against hallmark gene sets based on differentially expressed gene (DEG) ranking on bulk RNA-Seq revealed an enrichment in genes relating to inflammatory responses, including TNF signaling via NF-κB and the IL-6/JAK/STAT3 pathway (Supplemental Figure 4A) on D4 after symptom onset. Moreover, in the basal state, the STAT2-deficient patient displayed enhanced expression of the inflammatory signature at the transcriptome level relative to the control. Absolute cell-type deconvolution analysis demonstrated higher positive enrichment score for classical monocytes, basophils, CD8EM cells, and non-Vδ2 TCR-γδ T cells in the STAT2-deficient patient than in the control, both during acute COVID-19 illness and outside of infectious/inflammatory episodes (Supplemental Figure 4B). Given the absence of major immunophenotypic alterations in P10, the observed enrichment in these cell types probably reflects their (hyper)activation rather than their accumulation in peripheral blood. Neutrophils were the only cells for which the positive enrichment score in the STAT2 patient was much higher during acute mild COVID-19 than in the basal state or in a healthy control (Supplemental Figure 4B). This patient did not experience severe inflammation at the time of COVID-19, but our data nevertheless highlight an enhancement of inflammatory cascades (TNF/NF-κB and IL-6/JAK/STAT3) in STAT2 deficiency, both in the basal state and during acute COVID-19, mediated predominantly by neutrophils.

## Discussion

We describe inherited complete STAT2 deficiency in 23 patients of distant ancestries from 10 families in 7 countries. The predominant clinical presentation was disseminated infection after inoculation with LAV, especially the measles vaccine (clinical infection in 70% of the patients receiving any LAV). Disseminated disease after LAV vaccination for MMR has been reported in patients with other defects of type I IFN immunity, such as complete deficiencies of STAT1, IFNAR1, IFNAR2, and IRF9, but not deficiency of IL-10RB, a subunit of IFNLR (11, 20, 22–24, 45–49). The penetrance of disseminated disease following MMR in these patients was high but incomplete, except for patients with complete AR STAT1 deficiency. Patients with deficiencies of IFNAR1 or IFNAR2 are also susceptible to disease caused by the YFV LAV (24, 27). None of the patients with STAT2 deficiency reported here had been vaccinated against YFV. YFV vaccination is contraindicated for individuals with STAT1, STAT2, IRF9, IFNAR1, or IFNAR2 deficiencies. Live attenuated VZV vaccination has also caused disease in patients with STAT2 deficiency and is therefore also contraindicated. None of the known patients with an inborn error of type I/III IFN immunity had received the oral live attenuated poliovirus vaccine. It is probably safer to vaccinate these individuals with the inactivated poliovirus vaccine. Inborn errors of type I/III IFN immunity should be considered in any patient with adverse reactions to MMR, YFV, or VZV LAV. The presence of neutralizing anti–type I IFN autoantibodies should also be investigated (27).

Susceptibility to severe natural viral diseases was noted in 43% of STAT2-deficient patients (10/23). A high prevalence of influenza pneumonia was observed (6/7 or 86% of patients with PCR-proven influenza A). AR IRF9 deficiency, AR IRF7 deficiency, AD TLR3, and AR IFNAR2 deficiency also underlie influenza pneumonia (22, 23, 33, 50–53). All these defects affect both type I and type III IFN immunity through different mechanisms: TLR3 induces the transcription of type I and III IFNs; IRF7 is required for their amplification; and IRF9 is the DNA-binding partner in ISGF3 (54). AR STAT2 deficiency is the fifth monogenic etiology of critical influenza to be described (17). It may not be coincidental that these 4 etiologies impair both type I and type III IFN immunity, whereas critical influenza pneumonia has only been reported in 1 of the 29 known patients with IFNAR1 or IFNAR2 deficiency (19–21, 24–33) and in none of the more than 30 patients with IL10RB deficiency (55–58). Despite the high prevalence of herpesvirus infections in the general population and in STAT2-deficient patients, no life-threatening herpesvirus infections were reported in this cohort, besides 1 case of fatal HSE. No major anomalies in the immunophenotype or in antibody responses were detected in STAT2-deficient patients, in agreement with recent work evidencing normal antibody response to mRNA SARS-CoV-2 vaccines in the absence of type I IFN signaling (59). An overview of the infectious and immunologic phenotype of patients with AR complete STAT2, IFNAR1, IFNAR2, STAT1, IRF9, IRF7, and IL10RB deficiencies and patients with autosomal dominant or AR TLR3 deficiency is presented in Table 4.

The sample size is too limited to draw definitive conclusions on the nature and range of viral infections that cause life-threatening disease in STAT2 deficiency. We noted a predominance of severe infections with MMR vaccine, influenza, and herpesviruses. Moreover, most patients had infections with several other common viruses, including SARS-CoV-2, some without severe consequences. The diverse ancestries/countries of these patients suggest that these findings are genetically robust and largely independent of the viruses encountered. Our descriptions are reminiscent of those for AR IRF9 deficiency and IFNAR1 or IFNAR2 deficiencies (19–33). They contrast with the findings for patients with complete STAT1 deficiency, who have an almost completely penetrant phenotype of severe infections caused by multiple viruses, including HSV-1, VZV, CMV, and HHV6 (16, 17, 22). The severity of STAT1 deficiency may be due to the additional deficit of type II IFNs, which does not in itself underlie any particular viral illnesses (10, 12), but may aggravate deficits of type I and type III IFNs. The phenotype of STAT1 and STAT2 deficiency is in part reflected in mice, in that both *Stat1^–/–^* and *Stat2^–/–^* mice display increased susceptibility to viral infection with increased mortality and indications of inflammation, although formal comparisons are challenging (2). The incomplete penetrance of life-threatening viral diseases in patients with STAT2 deficiency accounts for 15 of the 23 patients being still alive at ages of 3 to 38 years (median: 11 years). Mortality is prominent in childhood for many reasons: first, the timing of most LAVs and incidence of primary viral infections is in childhood; second, 6 of the patients received immunoglobulins after initial presentation; third, further functional development of T and B cell function could provide protection. Six STAT2-deficient patients died of heart failure in the context of febrile illness without identified pathogen. Whether this points to a failure to detect the disease-causing pathogen, to inflammatory cardiomyopathy/viral myocarditis, or to a hitherto unknown role for STAT2 besides host defense is unknown. Ten of the 15 surviving patients receive antiviral prophylaxis (acyclovir or immunoglobulins), and 5 patients have remained well as of this writing in the absence of prophylaxis.

Systemic inflammation and HLH have been reported in several children with defective IFN responses (11, 20, 25, 26, 31, 60). Systemic inflammation was detected in 7 of 23 STAT2-deficient patients and was mostly triggered by exposure to LAV (*n* = 5). HLH has been described in patients with complete STAT1, IFNAR1, or IFNAR2 deficiency, whereas only prolonged fever has been reported in patients with the rarer IRF9 deficiency. We propose a 2-phase model in which deficiencies of type I IFN immunity in the early stages of a viral infection lead to uncontrolled viral replication, with the activation and/or recruitment of tissular and circulating leukocytes leading to tissue and systemic inflammation. This 2-phase model of overwhelming inflammation is the core pathogenic mechanism for both hypoxemic COVID-19 pneumonia and multisystem inflammatory syndrome in children (MIS-C) (52, 61–64). Our data reveal a TNF/NF-κB and JAK/STAT3 signaling signature and roles for circulating monocytes, neutrophils, and CD8EM cells in inflammation in patients with STAT2 or related deficiencies. Given the overall decrease in signaling proximal and distal to IFNAR in STAT2 deficiency, the observed lack of USP18 expression and prolonged STAT1 phosphorylation probably play a minor role in the hyperinflammation observed in patients with AR STAT2 deficiency, particularly as similar inflammatory episodes occur in patients with STAT1, IFNAR1, and IFNAR2 deficiencies. Nevertheless, more data are needed to document this model further.

There is no standard treatment for AR STAT2 deficiency. Based on the results in patients with AR STAT1 complete deficiency, which is invariably lethal if not treated by HSCT, HSCT may be beneficial and possibly even curative in STAT2-deficient patients (11). However, it remains unclear whether the morbidity and mortality associated with STAT2 deficiency outweigh the intrinsic risks of HSCT. STAT2 deficiency–related mortality is strictly due to infectious or inflammatory complications, so early diagnosis and early intervention in the event of an acute infection could improve prognosis substantially. Antiviral medication should be initiated, if indicated, and a role for exogenous IFN-γ has been explored in the acute phase of infection in a patient with IFNAR1 deficiency with severe COVID-19 pneumonia (30). The early administration of anti–SARS-CoV-2 combined monoclonal antibody therapy in an IRF9-deficient patient resulted in the rapid clearance of viremia (65). High-dose immunoglobulins (0.8–2 g/kg) and corticosteroids can be used to manage inflammatory complications of viral infection (16). Moreover, regular immunoglobulin replacement therapy or acyclovir may prevent severe infections with naturally occurring viruses. In conclusion, STAT2 deficiency underlies severe viral diseases characterized by excessive inflammation due to impaired responses to type I IFN in the initial phase of infection. Half of the patients described in this cohort have survived into teenage years or adulthood. The range of viral susceptibility remains to be determined.

## Methods

For complete information on Methods, see Supplemental Methods.

### Data availability.

Raw data generated from next-generation sequencing in this study were deposited in the NCBI Sequence Read Archive (SRA BioProject PRJNA936917, PRJNA818002, PRJNA856671, PRJNA898284, PRJNA924565, PRJNA856671). See also Supplemental Methods.

### Statistics.

Descriptive statistics were used (e.g., mean and SEM in plots, as described in the figure legends). No measure of significance has been used in this paper. Pseudobulk principal component analysis and gene-set enrichment analysis were used for analyzing scRNA-Seq data.

### Study approval.

Patients from previous reports were recruited by contacting physicians, and new patients were recruited via international calls and from the Human Genetics of Infectious Diseases Laboratory cohort of patients with severe viral infections. All patients or their legal guardians gave consent for participation in the study. The study was approved by the Ethical Research Committee of Leuven University Hospitals (protocol number S60905). The data were collected via a case record form.

## Author contributions

IM, JLC, QZ, and LM designed the research project. GB, LM, MO, DR, DM, M Momenilandi, NK, NHF, CJAD, FG, VB, and EJ conducted the experimental work. GB, LM, MO, DR, M Momenilandi, QZ, VB, CJAD, SBD, SYZ, and IM analyzed the data. GB, LM, and IM drafted the manuscript. GB, CJAD, CMC, ERT, MAS, BKAS, AL, RDL, M Meignien, AB, IB, AS, AR, S Hackett, LMB, MCM, MFA, JC, RWM, BJF, SYZ, S Hambleton, and IM provided patient care, recruited patients for the study, and provided patient data and samples. All the authors revised the manuscript. Authorship order was assigned based on relative contribution to the study, with the persons most involved in the research first and the most experienced contributors last. The order of the co–first authors was determined by respective contributions in terms of writing the first draft, analysis, and experiments.

## Supplementary Material

Supplemental data

## Figures and Tables

**Table 3:**
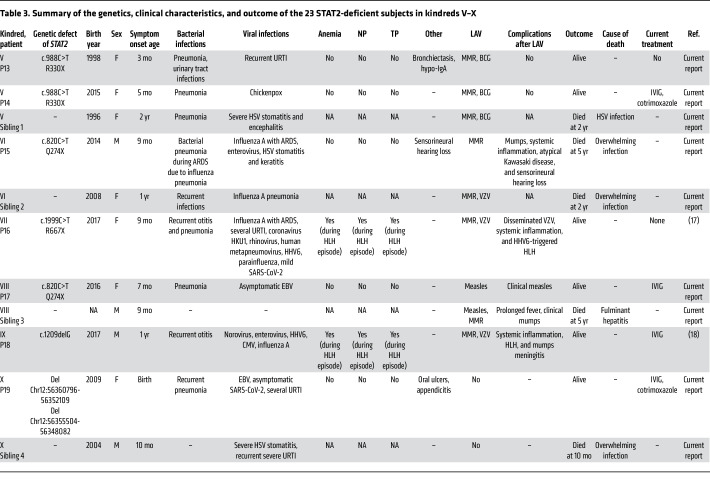
Summary of the genetics, clinical characteristics, and outcome of the 23 STAT2-deficient subjects in kindreds V–X

**Table 2:**
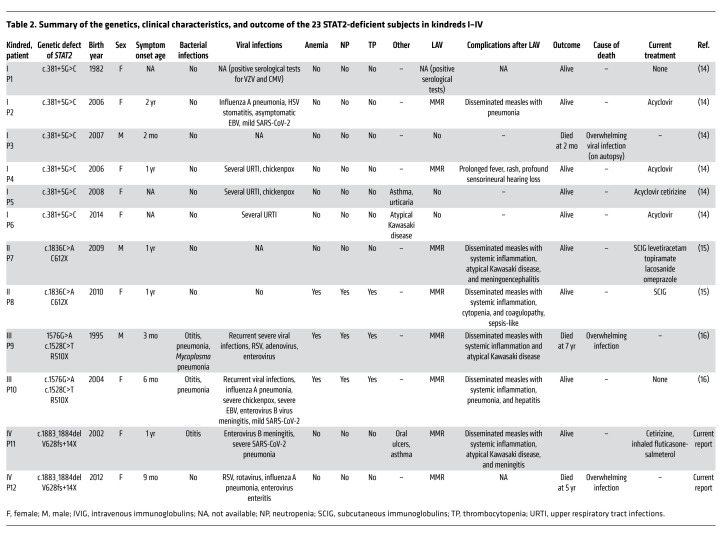
Summary of the genetics, clinical characteristics, and outcome of the 23 STAT2-deficient subjects in kindreds I–IV

**Table 1:**
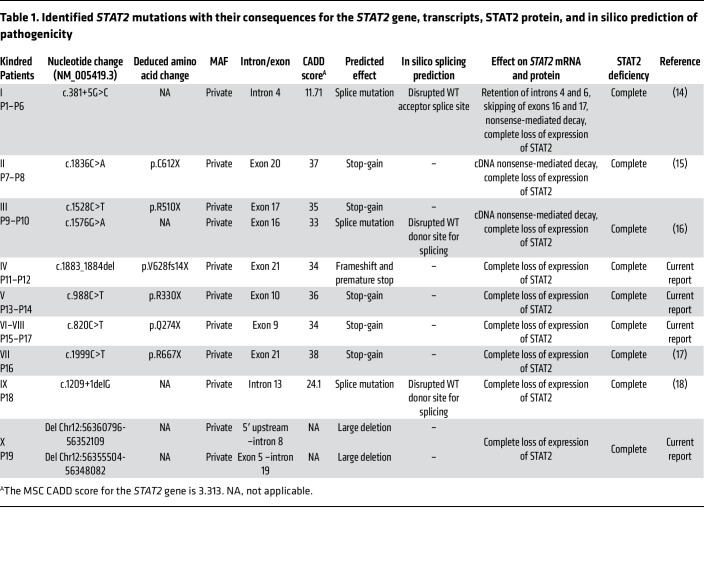
Identified *STAT2* mutations with their consequences for the *STAT2* gene, transcripts, STAT2 protein, and in silico prediction of pathogenicity

**Table 4:**
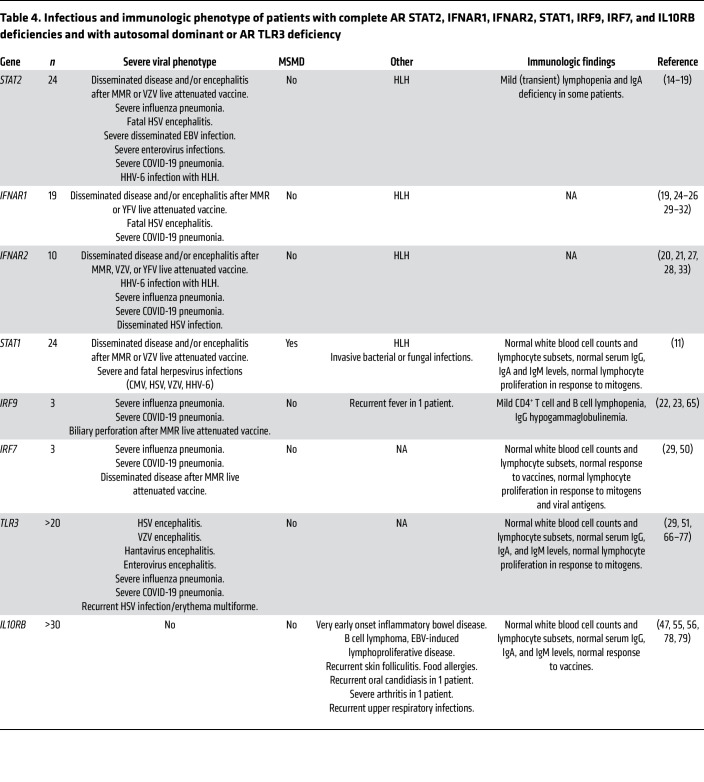
Infectious and immunologic phenotype of patients with complete AR STAT2, IFNAR1, IFNAR2, STAT1, IRF9, IRF7, and IL10RB deficiencies and with autosomal dominant or AR TLR3 deficiency

## Version 1 03/28/2023

In-Press Preview

## Version 2 06/15/2023

Electronic publication
