# Supplementary material for: Human inherited complete STAT2 deficiency underlies inflammatory viral diseases
Source: J Clin Invest. 2023 Jun 15;133(12):e168321. doi: 10.1172/JCI168321 (PMC10266780; doi:10.1172/JCI168321)
Supplement: Supplemental data [file jci-133-168321-s202.pdf]

Material and methods

Study of the STAT2 variants

Genetic sequencing

STAT2 mutations were identified by targeted Sanger sequencing in kindred I (P1-6) and by whole exome sequencing (WES)/targeted panel sequencing in all other patients. Additional variants are presented in supplemental Table S3.

Plasmids

The codon-optimized coding sequence for *STAT2* (NM_005419.4) was cloned into an untagged pCMV6 vector (Origene). Site-directed mutagenesis (NEB) was performed to obtain the indicated variant/mutant constructs. All constructs were re-sequenced (LCG Genomics) to ensure that no adventitious mutations were generated during cloning.

Transfection

HEK293T cells or STAT2 deficient fibrosarcoma cells (U6A) were transfected with either empty pCMV6 vector or with pCMV6-*STAT2* WT/mutant variants (100 ng/10^5 cells) in the presence of Lipofectamine 2000 reagent (Thermo Fisher Scientific) according to the manufacturer's protocol.

Evaluation of STAT1, STAT2 *and* USP18 *expression and STAT1 and STAT2 phosphorylation by immunoblotting*

EBV-transformed LCLs were generated according to standard protocols (1), from patients harboring the following genetic variants: for IFNAR1 deficiency: V225fs/W261X; for IFNAR2 deficiency: L79X/L79X; for STAT1 deficiency: c.1928insA/c.1928insA; for STAT2 deficiency: R510X/c.1576G>A (P10); for TYK2 deficiency: L767X/L767X, and for IRF9 deficiency: c.991G>A/c.991G>A.

For each set of conditions, 2*10^6 LCLs, with or without IFN-α2A pretreatment (10,000 U/mL, 1-6-24-48 hours, Miltenyi Biotec), were lysed in RIPA buffer (150 mM NaCl, 50 mM Tris, pH 8, 1% Triton X-100, 0.5% $C_{24}H_{39}NaO_4$, 0.1% SDS) supplemented with Pierce phosphatase inhibitor (Thermo Fisher Scientific) and complete protease inhibitor cocktail (Roche).

Transfected HEK293T cells, with or without IFN-α2A pretreatment (10,000 U/mL, 30 min, Miltenyi Biotec), were lysed in RIPA buffer (150 mM NaCl, 50 mM Tris, pH 8, 1% Triton X 100, 0.5% $C_{24}H_{39}NaO_4$, 0.1% SDS) supplemented with Pierce phosphatase inhibitor (Thermo Fisher Scientific) and complete protease inhibitor cocktail (Roche).

Protein lysates were subjected to SDS-PAGE and the resulting bands were transferred to a polyvinylidene fluoride membrane, which was probed with unconjugated primary antibodies [STAT1 (9167S, Cell Signaling Technology, 1/400), pSTAT1 (9167S (Tyr701), Cell Signaling Technology, 1/400), STAT2 (sc-514193, Santa Cruz Biotechnology, 1/200), pSTAT2 P-Y690 (AF2890 (Tyr689), R&D Systems, 1/200), and USP18 (4813S, Cell Signaling Technology, 1/500)] and HRP-conjugated secondary antibodies [goat anti-mouse (71045-3, Merck Life Science, 1/10,000) and mouse anti-rabbit (sc-2357, Santa Cruz Biotechnology, 1/10,000)]. An anti-β actin antibody (Sigma Aldrich, 1/9,000) was used as a loading control. SuperSignal West Pico Plus chemiluminescent substrate (STAT2, pSTAT2, USP18) and Pierce ECL western blotting substrate (STAT1, pSTAT1, β actin) (Thermo Fisher Scientific) were used to visualize HRP activity. Chemiluminescent signals were detected with a BioRad Imager and Image Lab 6.0.1 software was used for analysis.

Primary EBV-LCLs were generated from the cells of P19 according to standard protocols (1). 10^6 EBV-LCLs were stimulated with 1000 IU/ml IFN-α2B (Intron-A, Schering-Plough) for up to 24 hours and were then lysed [50 mM Tris-HCl (pH 7.5), 150 mM NaCl, 1% Nonidet P-40, 0.1% SDS, 0.5% sodium deoxycholate, 100 mM dithiothreitol (Sigma-Aldrich), 1× complete protease inhibitor cocktail (Roche, Basel, Switzerland), 1x PhosSTOP phosphatase inhibitors (Roche), 1× NuPAGE Loading Buffer (Life Technologies)]. Lysates were run on 4 -12% Tris-glycine polyacrylamide gels (Novex, Life Technologies) in 1× SDS NuPAGE MOPS Running Buffer (Life Technologies) with Prestained Plus Protein Ladder (Thermo Fisher Scientific) molecular weight markers. Proteins were transferred to polyvinyl difluoride membranes with 0.45 µm pores (Thermo Fisher Scientific) in NuPAGE Tris-glycine transfer buffer. The membranes were blocked by incubation in 5% bovine serum albumin (BSA) in Tris-buffered saline supplemented with 0.1%

Tween (TBS-T), and were washed several times before immunostaining by standard methods (incubation of the membranes with primary antibodies diluted in Tris-buffered saline 5% BSA overnight at 4°C, followed by several washings and incubation with secondary antibodies diluted in Tris-buffered saline 5% BSA for 1 hour at room temperature). Primary antibodies against the following proteins were used, with HRP-conjugated secondary antibodies (7074 and 7076, both from Cell Signaling Technology): STAT2 (sc-1668, Santa Cruz Biotechnology, 1/2,000), pSTAT2 (8841, Cell Signaling Technology, 1/2,000), USP18 (4813, Cell Signaling Technology, 1/1,000), and α-tubulin (3873, Cell Signaling Technology, 1/10,000). Membranes were developed with Immobilon Western Chemiluminescent HRP substrate (Millipore) and imaged on an LI-COR Odyssey Fc (LI-COR) imaging system.

RT-qPCR

Transfected U6A cells, with or without IFN-α2A pretreatment (10,000U/mL, 6 h, Miltenyi Biotec), were collected in Trizol Reagent (Ambion). RNA was extracted with the PureLink RNA mini kit (Thermo Fisher Scientific) and cDNA was generated with the SuperScript VILO cDNA synthesis kit (Thermo Fisher Scientific) according to manufacturers' protocols. We then performed qPCR for *STAT2*, *IFIT1*, *IFI27,* and *RSAD2* in SsoAdvanced Universal SYBR Green Supermix (BioRad) and ran the samples on a QuantStudio 3 system (Applied Biosystems). The results are expressed according to the ΔCt method, with GAPDH as the housekeeping gene, and data are normalized with respect to wild-type unstimulated conditions.

For each set of conditions, we collected $0.5*10^6$ LCLs, with or without IFN-α2A (10,000 U/mL, 1-6-24-48 hours, Miltenyi Biotec), or IFN-γ (1000 U/mL, 1-6-24-48 hours, R&D Systems) pretreatment, in Trizol Reagent (Ambion). RNA was extracted with the PureLink RNA mini kit (Thermo Fisher Scientific) and cDNA was generated with the SuperScript VILO cDNA synthesis kit (Thermo Fisher Scientific), according to manufacturers' protocols. We then performed qPCR for *STAT2*, *IFIT1*, *IFI27*, *RSAD2*, *IRF1*, *ICAM1, CIITA, SOCS1* and *SOCS3* in SsoAdvanced Universal SYBR Green Supermix (BioRad) and ran the samples on a QuantStudio 3 system (Applied Biosystems). The results are expressed according to the ΔCt method, with

GAPDH as the housekeeping gene, and data are normalized with respect to the healthy control unstimulated conditions.

RNA was extracted from primary EBV-LCLs from P19 with the ReliaPrep RNA Cell Miniprep System (Promega) and reverse-transcribed with the Superscript III polymerase (Thermo Fisher Scientific). The resulting cDNA templates were subjected to qPCR with TaqMan™ Gene Expression Master Mix (Applied Biosystems), according to manufacturer's instructions. Primers and related probes were designed by Roche Universal Probe Library System Assay Design (Roche, Basel, Switzerland). Plates were run on an AriaMx Real-time PCR System (Agilent Technologies). The results are expressed according to the ΔCt method, with the 18S rRNA gene as the housekeeping gene.

In vitro *susceptibility to viral infection*

SV40 fibroblasts were derived from dermal biopsy samples and immortalized by transformation with a plasmid-containing SV40 large T antigen according to standard protocols (2) from patients harboring the following genetic variants: IFNAR1 deficiency: Y481_insIHCGICFPVX/Y481_insIHCGICFPVX (3); STAT1 deficiency: c.1928insA/c.1928insA (4); STAT2 deficiency: R510X/c.1576G>A (P10) (5); IRF9 deficiency: c.991G>A/c.991G>A (6). We used $5*10^4$ SV40 fibroblasts per well to seed 48-well plates. The cells were infected, 16 hours later, with HSV-1 (KOS strain, ATCC), at a MOI of 0.001, in DMEM supplemented with 10% FBS. The cells were incubated in 250 µL of medium. For IFN-α2b pretreatment, the cells were incubated with 1000 IU/mL IFN-α2b for 16 hours before infection, and IFN-α2b was maintained in the medium throughout the entire course of HSV-1 infection. Cells and supernatants were collected at various time points after HSV-1 infection (12 h, 24 h, 48 h, 72 h) and analyzed by virus titration. HSV-1 titers were determined by calculating the 50% end point ($TCID_{50}$), as described by Reed and Muench (7), after the inoculation of Vero cell cultures.

Analysis of inflammatory responses in the patients

Cytokine levels

Serum was obtained, by centrifugation, from fresh whole-blood samples from a STAT2-deficient patient (P10) 6 months before COVID-19, on day 4 of COVID-19 and 5 months post-COVID-19, and eight healthy controls. The V-PLEX Proinflammatory panel 1 human kit (Meso Scale Discory) was used to measure the concentration of the following pro-inflammatory cytokines: IFN-γ, IL-1β, IL-2, IL-4, IL-6, IL-8, IL-10, IL-12p70, IL-13, TNF-α.

Bulk RNA sequencing

We performed mRNA sequencing with an Illumina NextSeq550, with a read length of 75 bp and a read depth of 170 M. All FASTQ sequences passed quality control and were aligned with the GRCh38 reference genome with STAR (2.6.1d). BAM files were converted to a raw count expression matrix with featurecount. Raw count data were normalized with DEseq2. The ensemble IDs targeting multiple genes were collapsed (averaged), and a final data matrix gene was generated for downstream analysis. Geneset enrichment analysis (GSEA) was conducted based on the fold-change ranking against the hallmark gene sets (http://www.gsea-msigdb.org/gsea/msigdb/genesets.jsp?collection=H). Immune system-related pathways are shown on a volcano plot. Three immune-related pathways are represented on heatmaps of RNA-seq-quantified gene expression (*z*-score-scaled $\log_2$ normalized counts) for the inflammatory response, TNF signaling via NF-κB and IL6-JAK-STAT3 signaling hallmark gene sets. Absolute cell-type deconvolution analysis was performed with published software (8). The absolute cell-type enrichment scores are presented as dot plots produced with library ggplot2 (https://cran.r-project.org/web/packages/ggplot2/index.html). The raw data generated from this study are deposited in the NCBI database under the NCBI-SRA BioProject ID PRJNA936917.

Single-cell RNA-sequencing analysis of leukocytes in the basal state and with IFN treatment

Single-cell RNA-sequencing (scRNASeq) analysis was performed on cryopreserved PBMCs, as previously described (9). Thawed PBMCs (filtered through a MACS SmartStrainer with 70-µm pores to remove large debris) were either directly analyzed or were incubated for six hours at 37°C, with or without

recombinant human IFN-α2B (1000 IU/ml), before analysis. Libraries were prepared with the Chromium Single-Cell Reagent Kit (v3 Chemistry) and sequenced with an Illumina NovaSeq 6000 sequencer. Sequences were preprocessed with CellRanger. Approximately 10,000 cells were sequenced per sample. Data were filtered manually on the basis of common quality-control metrics and integrated with Harmony (10). Two sequential graph-based clustering analyses were performed. The first round of clustering identified general leukocyte subsets, and the second round identified memory and effector T-lymphocyte subsets and NK lymphocytes with a sufficiently high resolution. Clusters were identified with the SingleR pipeline (11) guided by the normalized RNA-Seq dataset generated by Monaco et al. (8) together with manual inspection for cell type-specific marker genes. For baseline analysis, the CITE-Seq datasets obtained from the 10X Genomics web portal were also integrated to facilitate cluster identification. Raw data generated from this study are deposited in the NCBI database under the following NCBI-SRA projects: BioProject ID PRJNA818002, samples C229, C239, C427, C312; BioProject ID PRJNA856671, samples scRNASeq_Baseline_CTLJLC441_R1, scRNASeq_Baseline_CTLJLC441_R2, scRNASeq_Baseline_MB9159; BioProject ID PRJNA898284, samples SC3, SC4, SC5; BioProject ID PRJNA924565, samples scRNASeq_PBMC_CTLJLC441_Baseline_R3, scRNASeq_PBMC_CTLJLC368_Baseline; BioProject ID PRJNA856671, samples scRNASeq_CTLJLC368_NS_6h, scRNASeq_CTLJLC368_IFNa2_6h, scRNASeq_CTLJLC406_NS_6h, scRNASeq_CTLJLC406_IFNa2_6h, scRNASeq_MB30030_NS_6h, scRNASeq_MB30030_IFNa2_6h (9). The 10X datasets were then excluded from subsequent analyses. Gene expression was quantified at the single-cell level with Seurat (12). Pseudobulk analysis was performed by aggregating all reads from cells assigned to a given cluster, as previously described (13). We performed PCA on the read counts normalized through variance-stabilizing transformation with batch correction, using the removeBatchEffect function implemented in limma (14). Differential expression analysis was performed with DESeq2 (15). GSEA was conducted with the fgsea package, by projecting the fold-change ranking onto various MSigDB gene sets (http://www.gsea-msigdb.org/gsea/msigdb/genesets.jsp). Intercellular communication analysis was performed with

CellChat (16). WGCNA was performed in R (17). All analyses were performed in R v4 (http://www.R-project.org/).

Mass cytometry

Blood leukocyte subpopulation analysis was performed on 200 µL of fresh blood by deep immunophenotyping with mass cytometry (CyTOF), for a STAT2-deficient patient and a healthy control. CyTOF was performed on whole blood with an in-house panel (Supplemental Table S4), according to the manufacturer's instructions. Cells were frozen at −80°C after overnight iridium staining, and acquisition was performed on a Helios machine (Fluidigm). The samples were processed within 24 hours of sampling. Data analysis was performed with OMIQ software.

Supplemental figures

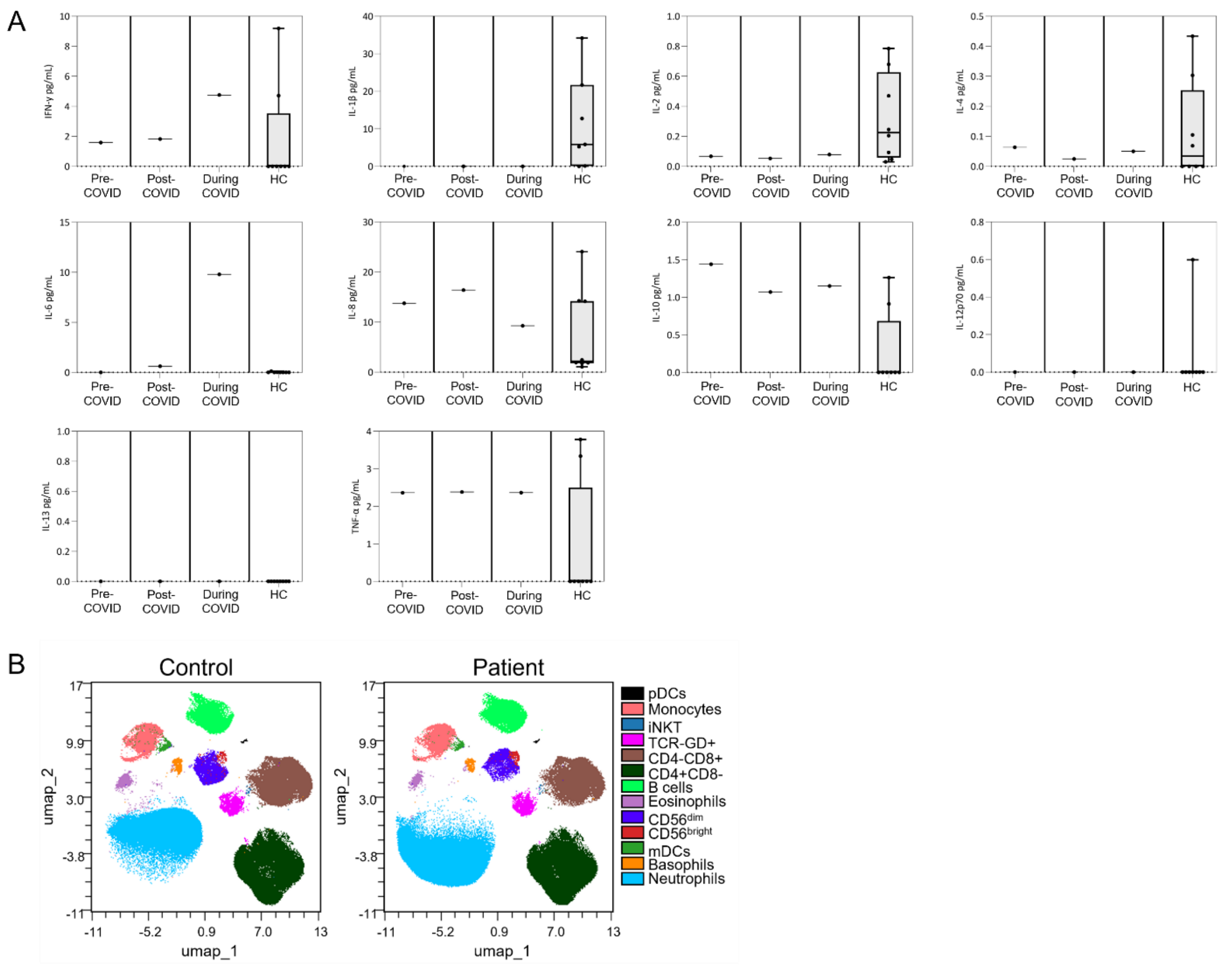

Supplemental Fig. S1. A) A human proinflammatory cytokine panel was used to measure the concentration of IFN-γ, IL-1β, IL-2, IL-4, IL-6, IL-8, IL-10, IL-12p70, IL-13, and TNF-α in serum samples from a STAT2 deficient patient (P10) in a state of well-being (Pre-COVID: at basal state; Post-COVID: 5 months after the infection) and during mild COVID-19 (n=1, 4 days from onset of symptoms) and from 8 healthy controls (HC). B) Immunophenotyping was performed on PBMCs derived from a STAT2 deficient patient (P10) aged 17 years. Uniform Manifold Approximation and Projection (UMAP) plots demonstrating the immunophenotype of PBMCs as determined by mass cytometry in a healthy control and the STAT2 deficient patient.

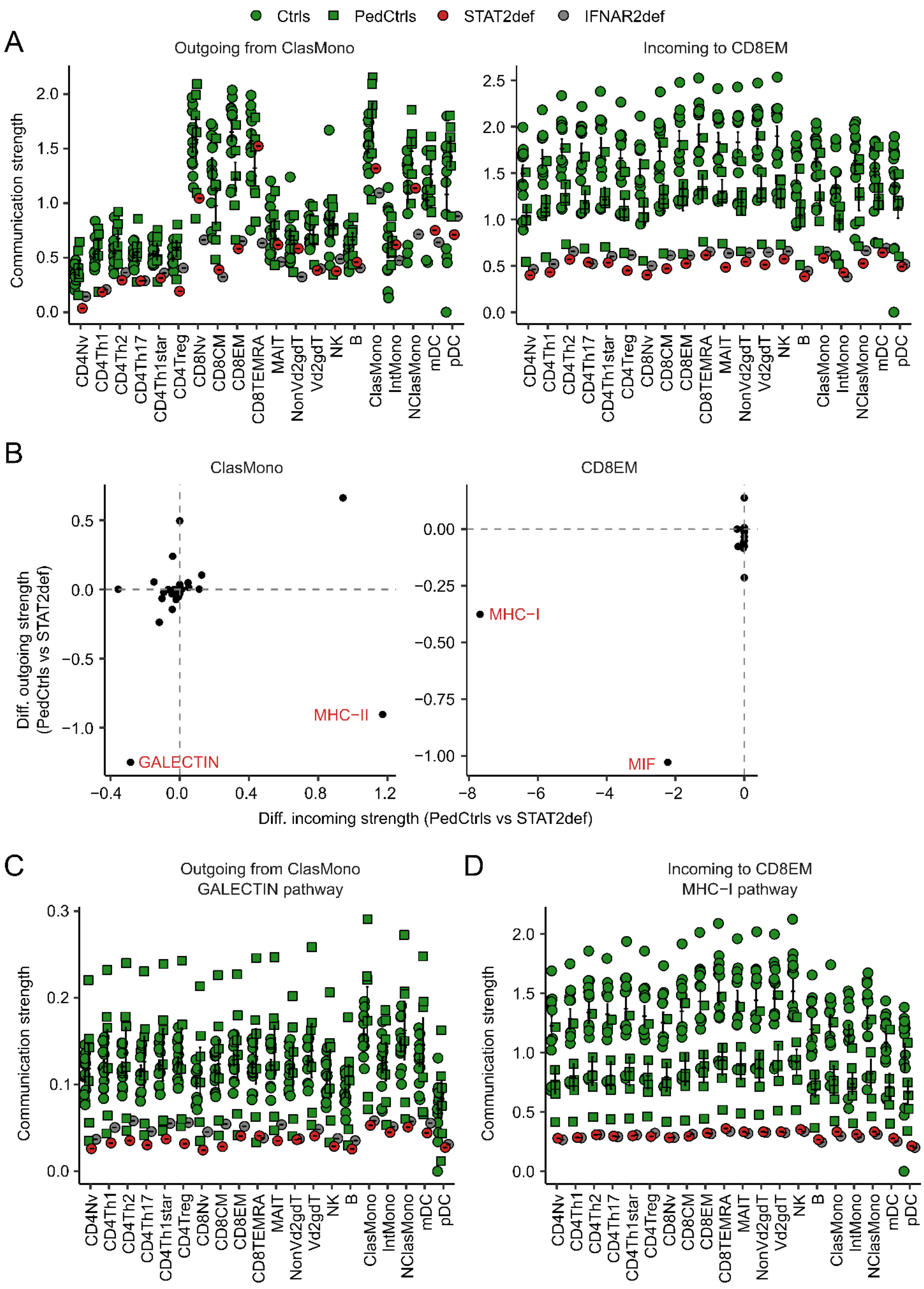

Supplemental Fig. S2. Deconvolution of inferred intercellular communications between leukocyte subsets with CellChat (Jin et al. Inference and analysis of cell-cell communication using CellChat. Nat Commun. 2021 Feb 17;12:1088.). Single-cell RNA sequencing data of PBMCs at baseline from healthy adult and pediatric controls, the STAT2 deficient patient (P10), and a patient with IFNAR2 deficiency were analyzed. A) Predicted interaction strength for communications outgoing from classical monocytes (left) or incoming to CD8 effector memory (EM) T cells (right). B) Differential pathway usage analysis. Pediatric controls and the STAT2 deficient patient were compared. C) Predicted outgoing signals involving the galectin pathway from classical monocytes. D) Predicted incoming signals involving the MHC class I pathway toward CD8 EM T cells.

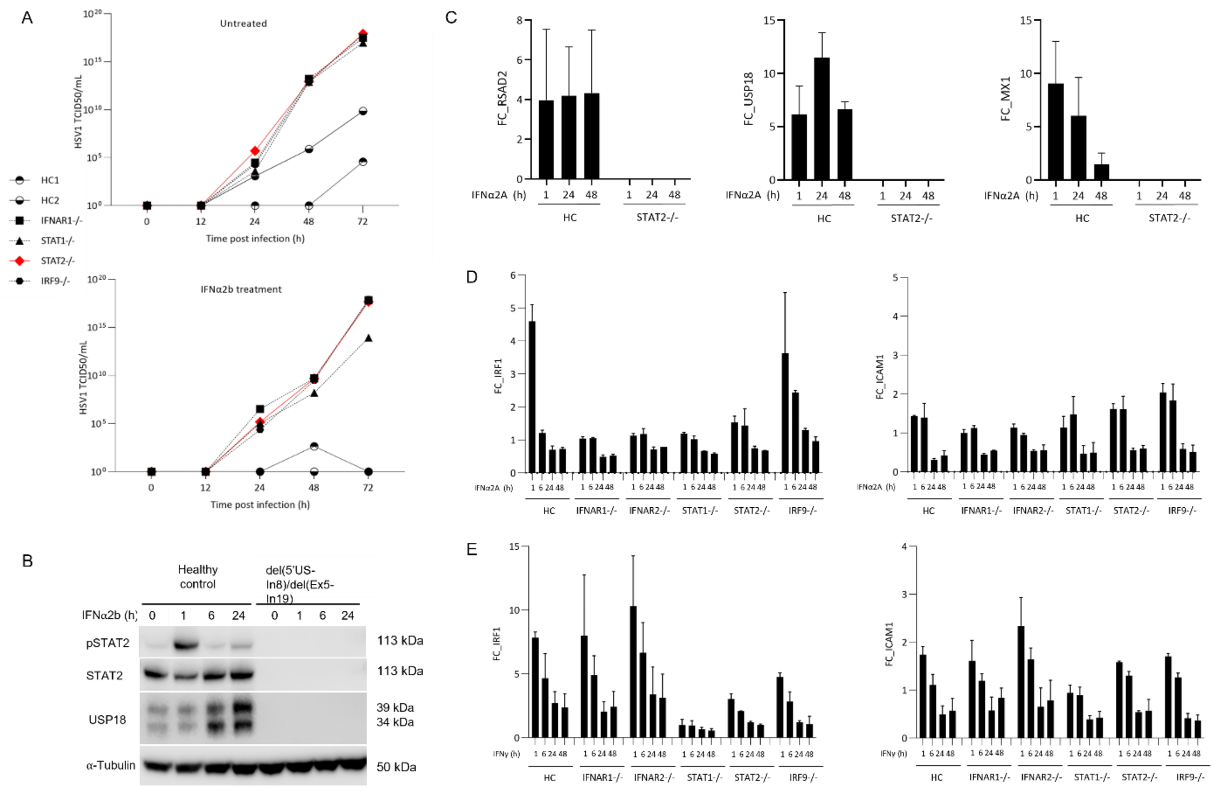

Supplemental Fig. S3. A) SV40 fibroblasts derived from either healthy controls or a patient with complete IFNAR1, STAT1, STAT2 or IRF9 deficiency were infected *in vitro* with HSV-1 at a MOI of 0.001, either without or with pre-treatment with 1,000 U/mL IFN-α2B for 16 hours before infection. The graph shows the viral titer in the different conditions 12, 24, 48 and 72 hours after infection. Viral titer was determined by calculation of the 50% end point (TCID50) after inoculation in Vero cell cultures. B) Immunoblot of phosphorylated STAT2, STAT2 and USP18 in primary EBV-LCL cells from a healthy control and P19, either in basal conditions (0) or after pretreatment with 1,000 U/mL IFN-α2B for 1, 6 or 24 hours, as indicated. One representative blot from three experiments is shown. C) Transcription levels of USP18, MX1 and RSAD2 assessed by RT-qPCR on primary EBV-LCL cells from healthy controls and P19, in basal conditions (0) or after pretreatment with 1,000 U/mL IFN-α2B for 1, 24 or 48 hours, as indicated. The mean (n = 3 or 4) and SEM are shown. Results are normalized relative to HC unstimulated conditions. D-E) Transcription levels for IRF1 and ICAM1 assessed by RT-qPCR on EBV-LCLs derived from either a healthy control (HC) or a patient with complete IFNAR1, IFNAR2, STAT1, STAT2 or IRF9 deficiency, after pretreatment with 10,000 U/mL IFN-α2A (D) or 1,000 U/mL IFN-γ (E) for 1, 6, 24 or 48 hours. HC: healthy control. The mean (n = 3) and SEM are shown. Results are normalized relative to HC unstimulated conditions.

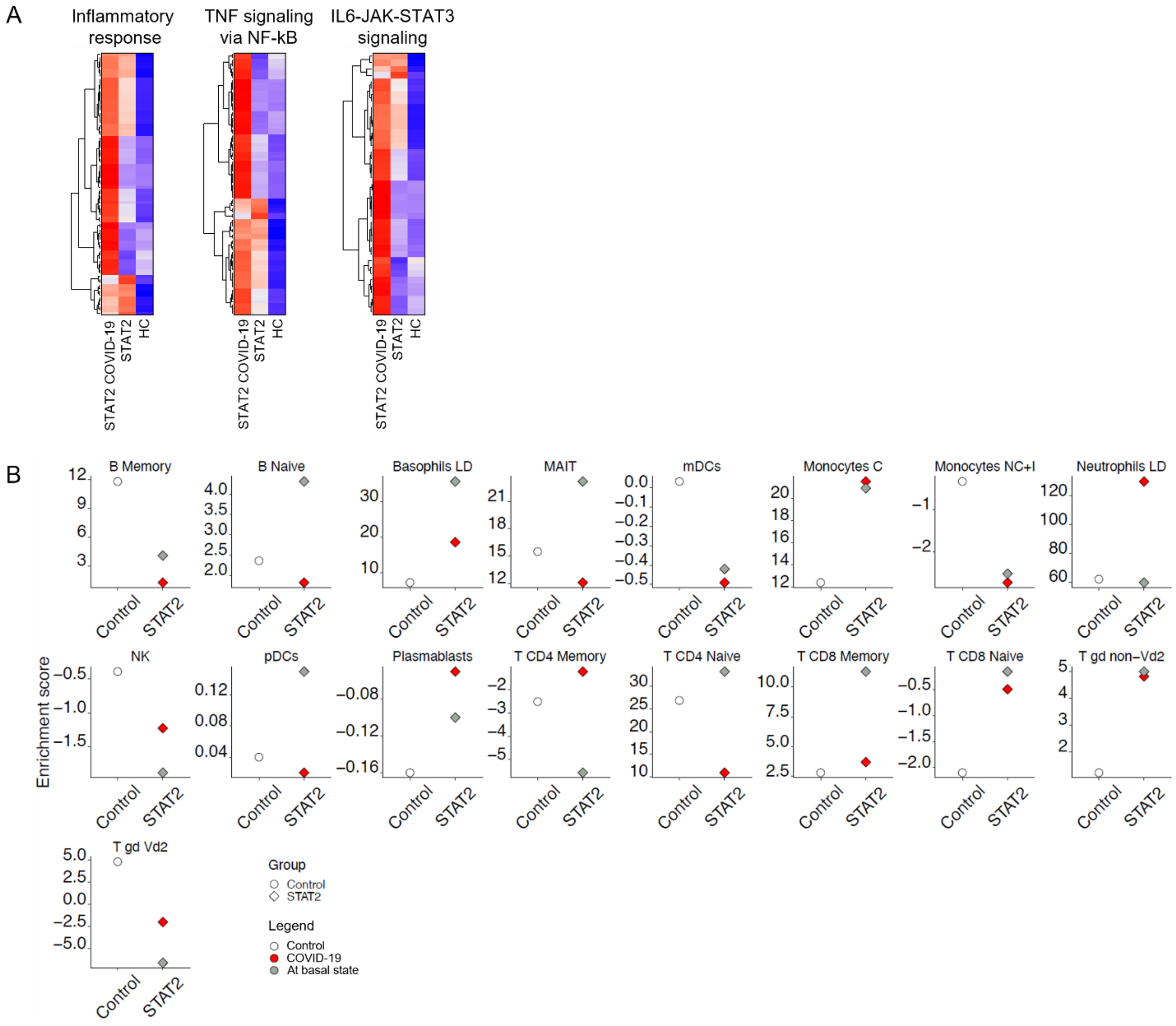

Supplemental Fig. S4. Bulk RNASeq of whole-blood leukocytes from a STAT2 deficient patient (P10) 4 days after the onset of symptoms due to acute SARS-CoV-2 infection (STAT2 COVID-19), the same STAT2 deficient patient outside infectious/inflammatory episodes (STAT2), and a healthy control (HC). A) Gene set enrichment analysis (GSEA) was conducted based on the fold-change of STAT2 COVID-19 or STAT2 versus HC, ranking against the Hallmark gene sets (http://www.gsea-msigdb.org/gsea/msigdb/genesets.jsp?collection=H). NES: normalized enrichment score. Three immune-related pathways are represented by heatmaps of RNAseq quantified gene expression (z-score scaled log2 normalized counts) for the Inflammatory response, TNF signaling via NF-kB and IL6-JAK-STAT3 signaling Hallmark gene sets. B) Absolute cell type deconvolution analysis was determined by using a published software (Monaco et al., Cell Rep. 2019 Feb 5;26(6):1627-1640.e7). The absolute cell type enrichment scores are presented by dot plot using library ggplot2 (https://cran.r-project.org/web/packages/ggplot2/index.html). COVID-19: during acute mild COVID-19 (D4 after onset); at basal state: 5 months after COVID-19.

Supplemental Table S1. VirScan results for P10 and P12: X represents a positive result of serological tests for the viruses in the first column.

**Virus**	**P10**	**P12**
Influenza A virus		
Influenza B virus		
Human respiratory syncytial virus		
Rhinovirus A	X	X
Rhinovirus B	X	
Rubella virus		X
Human metapneumovirus		
Norwalk virus		
Aichivirus A		
Enterovirus B	X	X
Enterovirus C		
Mamastrovirus 1	X	
Herpes simplex 1		
Herpes simplex 2		
Varicella-Zoster virus	X	
Epstein-Barr virus	X	
Cytomegalovirus		X
Human herpesvirus 6		
Human herpesvirus 7		
Human adenovirus B	X	
Human adenovirus C	X	

Supplemental Table S2. Immunologic results of 11 STAT2 deficient individuals.

	P2	P3	P7	P8	P9	P10	P11	P12	P15	P16	P17	P18	P19
**Kindred**	I	I	II	II	III	III	IV	IV	VI	VII	VIII	IX	X
**Variants**	c.381+5G>C	c.381+5G>C	C612X	C612X	R510X/ c.1576G>A	R510X/ c.1576G>A	V628fs14X	V628fs14X	Q274X	R667X	Q274X	c.1209+1delG (DelEx13)	Del(5'US-In8) Del(Ex6-In19)
**Age at testing**	1 y	1 m	11 m	1 y	3 y	1 y	1 y	1 y	1 y	1 y	1 y	1 y	10 y
**Total lymphocytes**	7270/μL (3600-8900)	4160/μL (3400-7600)	NA	1900/μL (3600-8900)	2300/μL (2300-5400)	1800/μL (3600-8900)	2974/μL (3600-8900)	8350/μL (3600-8900)	5540/μL (3600-8900)	3600/μL (3600-8900)	1580/μL (3600-8900)	6990/μL (3600-8900)	1820/μL (2000-2700)
**CD3+ T cells**	4781/μL – 65% (2100-6200 - 53-75)	3824/μL – 90% (2500-5500 – 53-84)	85% (49-76)	650/μL – 34% (2100-6200 – 53-75)	46% (56-75)	1173/μL – 64% (2100-6200 – 53-75)	1338/μL – 45% (2100-6200 – 53-75)	5770/μL – 69% (2100-6200 – 53-75)	3933/μL – 71% (2100-6200 – 53-75)	NA	1150/μL – 73% (2100-6200 – 53-75)	3651/μL – 51% (2100-6200 – 53-75)	1437 – 79% (1300-2000 – 66-76)
**CD4+ T cells**	3536/μL – 48% (1300-3400 - 32-51)	2937/μL – 70% (1600-4000 – 35-64)	35% (31-56%)	400/μL – 21% (1300-3400/μL 32-51%)	27% (28-47)	827/μL – 45% (1300-3400 – 32-51)	476/μL – 16% (1300-3400 – 32-51)	3750/μL – 45% (1300-3400 – 32-51)	2771/μL – 50% (1300-3400 – 32-51)	1008/μL – 28% (1300-3400 – 32-51)	590/μL – 37% (1300-3400 – 32-51)	1070/μL – 15% (1300-3400 – 32-51)	813/μL – 45% (700-1100 – 33-41)
**CD8+ T cells**	1207/μL – 16% (620-2000 - 14-30)	1047/μL – 25% (560-1700 – 12-28)	46% (12-24)	230/μL – 12% (620-2000 – 14-30)	17% (16-30)	309/μL – 17% (620-2000 – 14-30)	833/μL – 28% (620-2000 – 14-30)	2020/μL – 24% (620-2000 – 14-30)	1607/μL – 29% (620-2000 – 14-30)	756/μL – 21% (620-2000 – 14-30)	434/μL – 27% (620-2000 – 14-30)	2328/μL – 33% (620-2000 – 14-30)	474/μL – 26% (600-900 – 27-35)
**Naïve T cells**	31% (75-88)	NA	55% (76-89)	65% (75-88)	NA	NA	94% (75-88)	84% (75-88)	79% (75-88)	NA	NA	60% (75-88)	54% (35-72)
**T cell proliferation**													
**Antigens**	Normal	NA	NA	NA	↓	Normal	NA	NA	NA	NA	NA	Normal	NA
**Mitogens**	Normal	NA	Normal	Normal	Normal	Normal	NA	NA	NA	NA	NA	Normal	Normal
**NK cells**	1134/μL – 15% (180-920 – 3-15)	317/μL – 7% (170-1100 – 4-18)	3% (3-15)	230/μL – 12% (180-920 – 3-15)	NA	152/μL – 8% (180-920 – 3-15)	595/μL – 20% (180-920 – 3-15)	NA	665/μL – 11% (180-920 – 3-15)	216/μL – 6% (180-920 – 3-15)	327/μL – 20% (180-920 – 3-15)	1136/μL – 16% (180-920 – 3-15)	255 – 14% (163-515 – 4.8-15)
**CD19+ B cells**	727/μL – 10% (720-2600 – 16-35)	443/μL – 10% (300-2000/μL 6-32%)	9% (14-37)	860/μL – 45% (720-2600 – 16-35)	18% (14-33)	462/μL – 25% (720-2600 – 16-35)	625/μL – 21% (720-2600 – 16-35)	940/μL – 11% (720-2600 – 16-35)	609/μL – 10% (720-2600 – 16-35)	1368/μL – 38% (720-2600 – 16-35)	417/μL – 26% (720-2600 – 16-35)	2375/μL – 34% (720-2600 – 16-35)	127/μL – 7% (300-500 – 12-22)
**IgG**	7.53 g/L (3.83-10.7)	NA	9.66 g/L (3.83-10.7)	14.7 g/L* (3.83-10.7)	8.18 g/L (5.39-15)	8.02 g/L (3.83-10.7)	22 g/L (3.83-10.7)	5.32 g/L (3.83-10.7)	18 g/L* (3.83-10.7)	11.7 g/L* (3.83-10.7)	8.1 g/L* (3.83-10.7)	8.84 g/L (3.83-10.7)	12.5 g/L (8.7-11.7)
**IgA**	0.24 g/L (0.27-1.69)	NA	0.64 g/L (0.27-1.69)	0.17 g/L (0.27-1.69)	0.45 g/L (0.53-3.36)	1.22 g/L (0.27-1.69)	0.17 g/L (0.27-1.69)	0.58 g/L (0.27-1.69)	0.4 g/L (0.27-1.69)	0.4 g/L (0.27-1.69)	0.97 g/L (0.27-1.69)	0.52 g/L (0.27-1.69)	1.87 g/L (0.9-1.9)
**IgM**	0.58 g/L (0.28-1.13)	NA	0.57 g/L (0.28-1.13)	0.53 g/L (0.28-1.13)	0.67 g/L (0.26-1.06)	3.39 g/L (0.28-1.13)	3.75 g/L (0.28-1.13)	0.99 g/L (0.28-1.13)	1.52 g/L (0.28-1.13)	0.63 g/L (0.28-1.13)	1.84 g/L (0.28-1.13)	0.8 g/L (0.28-1.13)	1.04 g/L (0.8-1.9)

**IgE**	235 IU/ml	NA	NA	NA	NA	30 IU/ml	NA	NA	65 IU/ml	NA	NA	34 IU/ml	<2.33 IU/ml
**Antibodies against Tetanus vaccine**	4.8 IU/ml	NA	Normal	Normal	NA	Normal	NA	0.12 IU/ml	0.3 IU/ml	NA	7.51 mg/L	NA	NA
**Antibodies against pneumococcal vaccine**	528 IU/ml	NA	NA	Normal	NA	NA	NA	NA	NA	NA	NA	NA	NA
**Antibodies against poliovirus vaccine**	NA	NA	NA	NA	NA	Normal	NA	NA	NA	NA	NA	NA	NA
**Antibodies against HBV vaccine**	NA	NA	NA	NA	NA	Normal	NA	NA	NA	NA	NA	NA	NA

*during Ig supplementation. = normal, ↓ reduced. HBV: hepatitis B.

Reference ranges by age are given in brackets (18–20).

1. Hui-Yuen J, McAllister S, Koganti S, Hill E, Bhaduri-McIntosh S. Establishment of Epstein-Barr Virus Growth-transformed Lymphoblastoid Cell Lines. J Vis Exp. 2011 Nov 8;(57):3321.

2. Lisowska-Grospierre B, Fondaneche MC, Rols MP, Griscelli C, Fischer A. Two complementation groups account for most cases of inherited MHC class II deficiency. Human Molecular Genetics. 1994 Jun 1;3(6):953–8.

3. Bastard P, Manry J, Chen J, Rosain J, Seeleuthner Y, AbuZaitun O, et al. Herpes simplex encephalitis in a patient with a distinctive form of inherited IFNAR1 deficiency. J Clin Invest. 2021 Jan 4;131(1).

4. Chapgier A, Wynn RF, Jouanguy E, Filipe-Santos O, Zhang S, Feinberg J, et al. Human Complete Stat-1 Deficiency Is Associated with Defective Type I and II IFN Responses In Vitro but Immunity to Some Low Virulence Viruses In Vivo. The Journal of Immunology. 2006 Apr 15;176(8):5078–83.

5. Moens L, Van Eyck L, Jochmans D, Mitera T, Frans G, Bossuyt X, et al. A novel kindred with inherited STAT2 deficiency and severe viral illness. Journal of Allergy and Clinical Immunology. 2017 Jun 1;139(6):1995-1997.e9.

6. Hernandez N, Melki I, Jing H, Danielson J, Habib T, Huang S, et al. Life-threatening influenza pneumonitis in a child with inherited IRF9 deficiency. Journal of Experimental Medicine. 2018;In press.

7. Reed LJ, Muench H. A simple method of estimating fifty per cent endpoints. American Journal of Epidemiology. 1938 May 1;27(3):493–7.

8. Monaco G, Lee B, Xu W, Mustafah S, Hwang YY, Carré C, et al. RNA-Seq Signatures Normalized by mRNA Abundance Allow Absolute Deconvolution of Human Immune Cell Types. Cell Reports. 2019 Feb 5;26(6):1627-1640.e7.

9. Ogishi M, Arias AA, Yang R, Han JE, Zhang P, Rinchai D, et al. Impaired IL-23–dependent induction of IFN-γ underlies mycobacterial disease in patients with inherited TYK2 deficiency. Journal of Experimental Medicine. 2022 Sep 12;219(10):e20220094.

10. Korsunsky I, Millard N, Fan J, Slowikowski K, Zhang F, Wei K, et al. Fast, sensitive, and accurate integration of single cell data with Harmony. Nat Methods. 2019 Dec;16(12):1289–96.

11. Aran D, Looney AP, Liu L, Wu E, Fong V, Hsu A, et al. Reference-based analysis of lung single-cell sequencing reveals a transitional profibrotic macrophage. Nat Immunol. 2019 Feb;20(2):163–72.

12. Hao Y, Hao S, Andersen-Nissen E, Mauck WM, Zheng S, Butler A, et al. Integrated analysis of multimodal single-cell data. Cell. 2021 Jun 24;184(13):3573-3587.e29.

13. Crowell HL, Soneson C, Germain PL, Calini D, Collin L, Raposo C, et al. muscat detects subpopulation-specific state transitions from multi-sample multi-condition single-cell transcriptomics data. Nat Commun. 2020 Nov 30;11(1):6077.

14. Ritchie ME, Phipson B, Wu D, Hu Y, Law CW, Shi W, et al. limma powers differential expression analyses for RNA-sequencing and microarray studies. Nucleic Acids Research. 2015 Apr 20;43(7):e47.

15. Love MI, Huber W, Anders S. Moderated estimation of fold change and dispersion for RNA-seq data with DESeq2. Genome Biology. 2014 Dec 5;15(12):550.

16. Jin S, Guerrero-Juarez CF, Zhang L, Chang I, Ramos R, Kuan CH, et al. Inference and analysis of cell-cell communication using CellChat. Nat Commun. 2021 Feb 17;12:1088.

17. Langfelder P, Horvath S. WGCNA: an R package for weighted correlation network analysis. BMC Bioinformatics. 2008 Dec 29;9:559.

18. Shearer WT, Rosenblatt HM, Gelman RS, Oyomopito R, Plaeger S, Stiehm ER, et al. Lymphocyte subsets in healthy children from birth through 18 years of age: The pediatric AIDS clinical trials group P1009 study. Journal of Allergy and Clinical Immunology. 2003 Nov 1;112(5):973–80.

19. Buckley RH, Dees SC, O'Fallon WM. Serum Immunoglobulins: I. Levels in Normal Children and in Uncomplicated Childhood Allergy. Pediatrics. 1968 Mar 1;41(3):600–11.

20. Tosato F, Bucciol G, Pantano G, Putti MC, Sanzari M c., Basso G, et al. Lymphocytes subsets reference values in childhood. Cytometry. 2015 Jan 1;87(1):81–5.

Supplemental Table S3. Additional genetic results from the index patients of kindreds III to X. Analysis pipeline for exome results after quality control: 1) variants in known or predicted PID-related genes and de novo, compound heterozygous or homozygous variants in any gene are included; 2) variants with population frequency >2% are excluded; 3) only stoploss, stopgain, startloss, frameshift, inframe and non-synonymous variants are included; 4) synonymous variants excluded from step 3 are included in the analysis if they are exonic; 5) splice site variants excluded from step 3 and 4 are included in the analysis if they lie within 20 bp from a splice site; 6) all variants excluded so far are checked in The Human Gene Mutation Database (HGMD) and are included for analysis if present.

Patient	Type of analysis	Gene	Variant	CADD	MSC	Polyphen	SIFT	MAF	Inheritance	Disease
P10	Exome sequencing	*CTPS1*	c.820A>G, p.Ile274Val	14.91	2.7	Benign	Tolerated	0.00003	het	AR CID
		F7	c.739+7_739+80del	1.972	2.3	-	-	0.0002326	het	AR factor VII deficiency
		SRP54	c.973+7G>A	29.3	7.6	-	-	Private	het	AD severe congenital neutropenia
		KARS1	c.461T>C, p.Leu154Ser	17.23	-	Probably damaging	Deleterious	0.003559	het	AR Charcot-Marie-Tooth
		MAP3K14	c.2290A>G, p.Thr764Ala	16.96	-	Benign	Tolerated	0.000242	het	AR CID (NIK deficiency)
		MYO5B	c.5449A>G, p.Met1817Val	24.3	16.5	Benign	Tolerated	0.00006	het	AR congenital diarrhea
		IFIH1	c.1120C>T, p.Arg374Cys	23.1	17.4	Possibly damaging	Deleterious	0.0003696	het	AD Aicardi-Goutieres syndrome, AR viral sensing defect
		CFI	c.1019T>C, p.Ile340Thr	21.5	0	Possibly damaging	Deleterious	0.0002224	het	AR complement factor I deficiency
		DNAH11	c.9499A>C, p.Thr3167Pro	1.972	8	Benign	Tolerated	0.0002326	het	AR PCD
		TMEM66 (SARAF)	c.701-3_701-2insCCAGAGA TTCACCAACTCAGCAGGACC TCCTCCCCCAGGCTTTAAGT CTGAGTTCAC	-	-	-	-	Private	het	NA
		ABCB7	c.2005A>G, p.Thr669Ala	25.4	22.2	Possibly damaging	Deleterious	25.4	het	XL anemia with ataxia
		MGAT5B	c.322G>A, p.Ala108Thr<br>c.2071G>T, p.Ala691Ser	8.761<br>23	2.3	Benign<br>Benign	Tolerated<br>Deleterious	0.005<br>0.0002692	comp het	NA
		EPPK1	c.15196C>G, p.Leu5066Val<br>c.4200_4202del, p.Asn1400del	0.019<br>14.55	2.3	Benign<br>-	Tolerated<br>-	Private<br>0.004019	comp het	NA
		PRPF3	c.-48-5_-48-3dup	2.907	26.2	-	-	Private	hom	AR retinitis pigmentosa
		MEOX2	c.225_230del, p.His79_His80del	21.9	7.5	-	-	0.01002	hom	NA
P12	Exome sequencing	*TTC7A*	c.517+3A>G	21.7	5.6	-	-	0.001111	het	AR intestinal atresia
		EPCAM	c.-149+7T>C	11.09	5.8	-	-	0.01582	hom	AR congenital diarrhea
		DNASE1L3	c.127G>T, p.Asp43Tyr	28.1	21.3	Probably damaging	Deleterious	Private	het	AR SLE

		TLR3	c.1521C>A, p.Asn507Lys	21.7	22.4	Possibly damaging	Deleterious	Private	het	AR/AD herpes simplex encephalitis, influenza pneumonia
		TRAF3IP2	c.911_912del, p.Val304GlufsTer46	.	4.7	-	-	Private	het	AR CMC
		DNAH11	c.8809G>A, p.Asp2937Asn	24.6	8	Benign	Deleterious	0.003165	hom	AR PCD
		GIMAP6	c.1-10_1-5del	0.493	2.7	-	-	0.000018	het	NA
		EXTL3	c.-569-12T>G	6.623	23.7	-	-	0.0129	het	AR immunoskeletal dysplasia with neurodevelopmental abnormalities
		ADAMTS13	c.1261C>T, p.Arg421Cys	32	7.4	Possibly damaging	Deleterious	0.001796	het	AR TTP
		FANCF	c.647G>C, p.Arg216Pro	12.10	19.9	Benign	Tolerated	0.0043	het	AR Fanconi anemia
		CRACR2A	c.1367G>C, p.Gly456Ala	1.327	NA	Benign	Tolerated	Private	het	AR CID
		POLE	c.2865-5_2865-4dup<br>c.2089C>G, p.Pro697Ala	2.131<br>0.111	5.5	-<br>Benign	-<br>Tolerated	0.003335<br>0.001735	hom<br>het	AR malformative syndromes with immunodeficiency (FILS, IMAGEI)
		RNASEH2B	c.64+8C>T	9.270	25.4	-	-	0.00641	het	AR Aicardi-Goutieres syndrome
		DNASE1	c.499G>C, p.Asp167His	22.5	15.3	Probably damaging	Deleterious	0.0004808	het	AD SLE
		CREBBP	c.4133+4A>G	9.114	17.3	-	-	0.003999	het	AD malformative syndromes (Menke-Hennekam, Rubinstein-Taybi)
		PSMB10	c.470C>T, p.Ser157Phe	32	16.5	Probably damaging	Deleterious	0.0002882	het	AR autoinflammatory syndrome
		PTPN2	c.70-10T>C	19.08	4.6	-	-	0.0001519	het	AD very early onset IBD
		RELB	c.870G>C, p.Glu290Asp	17.77	5.6	Benign	Tolerated	0.0023	het	AR CID
		DOCK11	c.2878-4dup	0.465	1.9	-	-	0.004847	hom	NA
P14	Exome sequencing	*MOGS*	c.1604G>A, p.Arg535Gln	14.03	2.5	Benign	Tolerated	0.001	het	AR CDG
		FAT4	c.10750C>G, p.Leu3584Val	21.9	6.1	Probably damaging	Deleterious	0.0004776	het	AR Hennekam lymphangiectasia-lymphedema syndrome
		DOCK2	c.4201G>T, p.Ala1401Ser	18.71	28.8	Benign	Tolerated	0.0053	het	AR SCID
		DNAAF5	c.1000A>C, p.Thr334Pro<br>c.1010A>C, p.His337Pro	1.850<br>16.71	24.1	Benign<br>Benign	Tolerated<br>Deleterious	0.00834<br>0.0007776	het<br>het	AR PCD
		FANCC	c.178G>A, p.Val60Ile	0.015	7.5	Benign	Tolerated	0.00191	het	AR Fanconi anemia
		MYO5A	c.3858+7A>T	12.79	16.1	-	-	0.0009881	het	AR Griscelli syndrome
		HYDIN	c.7483G>A, p.Asp2495Asn	17.92	4.4	Benign	Deleterious	0.006329	het	AR PCD
		ITPKC	c.1760G>A, p.Gly587Glu	22.7	4.8	Benign	Deleterious	Private	het	NA
		PI4KA	c.4838C>T, p.Ala1613Val	29.9	23.6	Possibly	Deleterious	0.000956	het	AR intestinal atresia

						damaging				
P15	Mendeliome	*B3GALT6*	c.556T>C, p.Phe186Leu	32	22	Possibly damaging	Tolerated	0.00023	het	AR Al-Gazali syndrome, Ehlers-Danlos syndrome
		CC2D2A	c.3364C>T, p.Pro1122Ser	25.7	9.9	Probably damaging	Deleterious	0.000004	het	AR malformative syndromes (COACH, Meckel, Joubert)
P16	PID gene panel	*DOCK8*	c.54-1G>T						het	AR hyper-IgE syndrome
		IL2RA	c.263G>A, p.Arg88Gln						het	AR CD25 deficiency
		PRF1	c.356G>A, p.Arg119Gln						het	AR familial hemophagocytic lymphohistiocytosis
		RMRP	NR_003051.3:n.-31A>G						het	AR cartilage-hair hypoplasia
		SEMA3E	c.322A>C, p.Lys108Gln						het	AD CHARGE syndrome
P17	PID gene panel	*STXBP2*	c.1421C>T, p.Pro474Leu	24.9	7.2	Probably damaging	Deleterious	0.000004	het	AR familial hemophagocytic lymphohistiocytosis with severe enteropathy
P18	PID gene panel	*CARD9*	c.1277A>G, p.Asp426Gly	24.1	10.2632 4612	Possibly damaging	Deleterious	0.000064	het	AR chronic mucocutaneous candidiasis
	Whole-genome oligonucleotide array CGH	arr[GRCh37] 15q25.3(86305124_87572261)x3								NA
P19	Exome sequencing	*NLRP3*	c.846C>A, p.Asn282Lys	7.112	2.2	Benign	Tolerated	Private	het	AD autoinflammatory syndromes (CINCA Muckle-Wells, familial cold autoinflammatory syndrome)
		SERPING1	c.283A>C, p.Thr95Pro<br>c.286A>C, p.Thr96Pro	1.095<br>0.238	4.9	Benign<br>Benign	Tolerated<br>Tolerated	0.000046<br>Private	het<br>het	C1 inhibitor deficiency, hereditary angioedema
		MVK	c.793C>G, p.Leu265Val	22.3	11.2	Probably damaging	Deleterious	Private	het	AR hyper-IgD syndrome
		CARD14	c.658C>T, p.Arg220Cys	25.4	11.9	Possibly damaging	Deleterious	Private	het	AD psoriasis
		EPG5	c.2718+3A>G	21.2	11.7	-	-	0.0003913	het	AR VICI syndrome
		ADGRE2	c.1137G>C, p.Gln379His	14.26	-	Possibly damaging	Tolerated	0.000026	het	AD vibratory urticaria

		JAGN1	c.250C>G, p.Pro84Ala	24.8	20.7	Probably damaging	Deleterious	Private	het	AR severe congenital neutropenia
		MASP1	c.1931C>T, p.Thr644Met	27.5	16.8817 5735	Probably damaging	Deleterious	0.002084	het	AR 3MC syndrome
		ALPK1	c.3499G>A, p.Glu1167Lys	29.0	2.4	Probably damaging	Deleterious	Private	het	AD ROSAH syndrome
		IL6ST	c.1841-5_1841-4del	8.148	4	-	-	Private	het	Hyper-IgE syndrome
		IKZF1	c.761G>T, p.Cys254Phe	24.5	25.4	Possibly damaging	Tolerated	Private	het	AD CVID
		MCM7	c.277-16del	0.060	24	-	-	Private	het	AR Meier-Gorlin syndrome, lipodystrophy and adrenal insufficiency
		CDH17	c.928G>A, p.Ala310Thr	26.5	2.3	Probably damaging	Deleterious	0.0002918	het	AR SCID
		VPS13B	c.5520G>C, p.Lys1840Asn	16.44	15.3	Benign	Tolerated	Private	het	AR Cohen syndrome

AD: autosomal dominant; AR: autosomal recessive; CADD: combined annotation-dependent depletion; CDG: Congenital disorder of glycosylation; CID: combined immunodeficiency; CMC: chronic mucocutaneous candidiasis; comp het: compound heterozygous; CVID: common variable immunodeficiency; het: heterozygous; hom: homozygous; IBD: inflammatory bowel disease; MSC: mutation significance cutoff; NA: not applicable; PCD: primary ciliary dyskinesia; PID: primary immunodeficiency; SCID: severe combined immune deficiency; SIFT: sorting intolerant from tolerant; SLE: systemic lupus erythematosus; TTP: thrombotic thrombocytopenic purpura; XL: X linked.

Table S4. Antibodies used for mass cytometry (CyTOF) on fresh whole blood.

Metal	Target	Clone	Manufacturer	Catalog
**163Dy**	CXCR3	G025H7	Fluidigm	3163004B
**152Sm**	TCRgd	11F2	Fluidigm	3152008B
**142Nd**	CD19	HIB19	Fluidigm	3142001B
**144Nd**	CD38	HIT2	Fluidigm	3144014B
**151Eu**	CD123	6H6	Fluidigm	3151001B
**153Eu**	Va7.2	3C10	Fluidigm	3153024B
**154Sm**	CD3	UCHT1	Fluidigm	3154003B
**155Gd**	CD45RA	HI100	Fluidigm	3155011B
**158Gd**	CD27	L128	Fluidigm	3158010B
**159Tb**	CD1c	L161	Biolegend	331502
**161Dy**	CLEC9A	8F9	Fluidigm	3161018B
**164Dy**	CD161	HP-3G10	Fluidigm	3164009B
**168Er**	CD8	SK1	Fluidigm	3168002B
**170Er**	iNKT	6B11	Fluidigm	3170015B
**175Lu**	CCR4	L291H4	Fluidigm	3175035A
**174Yb**	CD4	RPA-T4	Biolegend	300502
**162Dy**	CD21	REA940	Miltenyi Biotec Inc.	130-124-315
**165Ho**	NKG2C	REA205	Miltenyi Biotec Inc.	130-122-278
**148Nd**	CD20	2H7	Biolegend	302302
**173Yb**	HLA-DR	L243	Fluidigm	3173005B
**156Gd**	CCR10	REA326	Miltenyi Biotec Inc.	130-122-317
**089Y**	CD45	HI30	Fluidigm	3089003B
**116Cd**	CD66b	QA17A51	Biolegend	396902
**141Pr**	CCR6	G034E3	Fluidigm	3141003A
**143Nd**	CD127	A019D5	Fluidigm	3143012B
**147Sm**	CD11c	Bu15	Fluidigm	3147008B
**149Sm**	CD25	2A3	Fluidigm	3149010B
**150Nd**	NKVFS1	NKVFS1	Bio Rad	MCA2243GA
**167Er**	CCR7	G043H7	Fluidigm	3167009A
**169Tm**	NKG2A	Z199	Fluidigm	3169013B
**171Yb**	CXCR5	RF8B2	Fluidigm	3171014B
**166Er**	CD24	ML5	Fluidigm	3166007B

**145Nd**	CD31	WM59	Fluidigm	3145004B
**160Gd**	CD14	M5E2	Fluidigm	3160001B
**176Yb**	CD56	NCAM16.2	Fluidigm	3176008B
**172Yb**	CD57	HNK-1	Biolegend	359602
**150Nd**	KIR3DL1L2	REA970	Miltenyi Biotec Inc.	130-126-489
**146Nd**	IgD	IA6-2	Fluidigm	3146005B
**209Bi**	CD16	3G8	Fluidigm	3209002B

Bibliography

1. Hui-Yuen J, McAllister S, Koganti S, Hill E, Bhaduri-McIntosh S. Establishment of Epstein-Barr Virus Growth-transformed Lymphoblastoid Cell Lines. J Vis Exp. 2011 Nov 8;(57):3321.

2. Lisowska-Grospierre B, Fondaneche MC, Rols MP, Griscelli C, Fischer A. Two complementation groups account for most cases of inherited MHC class II deficiency. Human Molecular Genetics. 1994 Jun 1;3(6):953–8.

3. Bastard P, Manry J, Chen J, Rosain J, Seeleuthner Y, AbuZaitun O, et al. Herpes simplex encephalitis in a patient with a distinctive form of inherited IFNAR1 deficiency. J Clin Invest. 2021 Jan 4;131(1).

4. Chapgier A, Wynn RF, Jouanguy E, Filipe-Santos O, Zhang S, Feinberg J, et al. Human Complete Stat-1 Deficiency Is Associated with Defective Type I and II IFN Responses In Vitro but Immunity to Some Low Virulence Viruses In Vivo. The Journal of Immunology. 2006 Apr 15;176(8):5078–83.

5. Moens L, Van Eyck L, Jochmans D, Mitera T, Frans G, Bossuyt X, et al. A novel kindred with inherited STAT2 deficiency and severe viral illness. Journal of Allergy and Clinical Immunology. 2017 Jun 1;139(6):1995-1997.e9.

6. Hernandez N, Melki I, Jing H, Danielson J, Habib T, Huang S, et al. Life-threatening influenza pneumonitis in a child with inherited IRF9 deficiency. Journal of Experimental Medicine. 2018;In press.

7. Reed LJ, Muench H. A simple method of estimating fifty per cent endpoints. American Journal of Epidemiology. 1938 May 1;27(3):493–7.

8. Monaco G, Lee B, Xu W, Mustafah S, Hwang YY, Carré C, et al. RNA-Seq Signatures Normalized by mRNA Abundance Allow Absolute Deconvolution of Human Immune Cell Types. Cell Reports. 2019 Feb 5;26(6):1627-1640.e7.

9. Ogishi M, Arias AA, Yang R, Han JE, Zhang P, Rinchai D, et al. Impaired IL-23–dependent induction of IFN-γ underlies mycobacterial disease in patients with inherited TYK2 deficiency. Journal of Experimental Medicine. 2022 Sep 12;219(10):e20220094.

10. Korsunsky I, Millard N, Fan J, Slowikowski K, Zhang F, Wei K, et al. Fast, sensitive, and accurate integration of single cell data with Harmony. Nat Methods. 2019 Dec;16(12):1289–96.

11. Aran D, Looney AP, Liu L, Wu E, Fong V, Hsu A, et al. Reference-based analysis of lung single-cell sequencing reveals a transitional profibrotic macrophage. Nat Immunol. 2019 Feb;20(2):163–72.

12. Hao Y, Hao S, Andersen-Nissen E, Mauck WM, Zheng S, Butler A, et al. Integrated analysis of multimodal single-cell data. Cell. 2021 Jun 24;184(13):3573-3587.e29.

13. Crowell HL, Soneson C, Germain PL, Calini D, Collin L, Raposo C, et al. muscat detects subpopulation-specific state transitions from multi-sample multi-condition single-cell transcriptomics data. Nat Commun. 2020 Nov 30;11(1):6077.

14. Ritchie ME, Phipson B, Wu D, Hu Y, Law CW, Shi W, et al. limma powers differential expression analyses for RNA-sequencing and microarray studies. Nucleic Acids Research. 2015 Apr 20;43(7):e47.

15. Love MI, Huber W, Anders S. Moderated estimation of fold change and dispersion for RNA-seq data with DESeq2. Genome Biology. 2014 Dec 5;15(12):550.

16. Jin S, Guerrero-Juarez CF, Zhang L, Chang I, Ramos R, Kuan CH, et al. Inference and analysis of cell-cell communication using CellChat. Nat Commun. 2021 Feb 17;12:1088.

17. Langfelder P, Horvath S. WGCNA: an R package for weighted correlation network analysis. BMC Bioinformatics. 2008 Dec 29;9:559.

18. Shearer WT, Rosenblatt HM, Gelman RS, Oyomopito R, Plaeger S, Stiehm ER, et al. Lymphocyte subsets in healthy children from birth through 18 years of age: The pediatric AIDS clinical trials group P1009 study. Journal of Allergy and Clinical Immunology. 2003 Nov 1;112(5):973–80.

19. Buckley RH, Dees SC, O'Fallon WM. Serum Immunoglobulins: I. Levels in Normal Children and in Uncomplicated Childhood Allergy. Pediatrics. 1968 Mar 1;41(3):600–11.

20. Tosato F, Bucciol G, Pantano G, Putti MC, Sanzari M c., Basso G, et al. Lymphocytes subsets reference values in childhood. Cytometry. 2015 Jan 1;87(1):81–5.

Members of the COVID Human Genetic Effort:

Laurent Abel[1,2,3], Andres Augusto Arias[4], Lisa Arkin[5], Alexandre Belot[6], Anastasia Bondarenko[7], Alessandro Borghesi[8], John Chrstodoulou[9], Roger Colobran[10], Clifton Dalgard[11], Xavier Marie Duval[12], Ali Amara[12], Hagit Baris Feldman[13], Jacques Fellay[14], Carlos Flores[15], Jose Lluis Franco[4], Sehime Gülsün Temel[16], Filomeen Haerynck[17], Rabi Halwani[18], Aurélie Cobat[1.2.3], Elena Hsie[19], Yuval Itan[20], Tom Le Voyer[1], Davood Mansour[21], France Mentre[22], Trine H Mogensen[23], Lisa Ng[24], Antonia Novelli[25], Satoshi Okada[26], Keisuke Okamoto[27], Rebeca Perez de Diego[28], Jordi Pérez Tur[29], David Perlin[30], Jonathan Peter[31], Cecilia Poli[32], Carolina Prando[33], Aurora Pujol Onofre[34], Lluis Quintana Murci[35], Laurent Renia[24], Jose Carlos Rodriguez Gallego[36], Anna Sediva[37], Mohammad Shahrooei[38], Ondrei Slaby[39], Andras Spaan[40], Ivan Tancevski[41], Stuart Tangye[42], Christian Thorball[43], Stuart Turvey[44], Furkan Uddin[45], Andreakos Vangelis[46], Sophie Assant[47], Alessandro Aiuti[48], Pere Soler-Palacin[49], Sara Espinosa[50], Yenan Bryceson[51], Don Vinh[52], Joost Wauters[53], Tayfun Ozcelik[54], Igor Resnik[55]

1. Laboratory of Human Genetics of Infectious Diseases, Necker Branch, INSERM U1163, Necker Hospital for Sick Children, Paris, France.
2. University of Paris, Imagine Institute, Paris, France.
3. St. Giles Laboratory of Human Genetics of Infectious Diseases, Rockefeller Branch, Rockefeller University, New York, NY, USA.
4. Group of Primary Immunodeficiencies, University of Antioquia UdeA, Medellin, Colombia.
5. Department of Dermatology, School of Medicine and Public Health, University of Wisconsin-Madison, Madison, WI, USA.
6. Pediatric Nephrology, Rheumatology, Dermatology, HFME, Hospices Civils de Lyon, National Referee Centre RAISE, and INSERM U1111, Université de Lyon, Lyon, France.
7. Department of Pediatrics, Immunology, Infectious and Rare Diseases, European Medical School, International European University (Kyiv, Ukraine)
8. Fondazione IRCCS Policlinico San Matteo di Pavia, Fellay Laboratory, Ecole Polytechnique Fédérale de Lausanne, Lausanne, Switzerland
9. Murdoch Children's Research Institute and Department of Paediatrics, University of Melbourne, Australia
10. Hospital Universitari Val d'Hebron, Barcelona, Spain
11. Department of Anatomy, Physiology & Genetics, Uniformed Services University of the Health Sciences, Bethesda, MD, USA
12. Université de Paris, IAME UMR-S 1137, INSERM, Paris, France; Inserm CIC 1425, Paris, France.
13. The Genetics Institute, Tel Aviv Sourasky Medical Center and Sackler Faculty of Medicine, Tel Aviv University, Tel Aviv, Israel
14. School of Life Sciences, Ecole Polytechnique Fédérale de Lausanne, Lausanne, Switzerland; Precision Medicine Unit, Lausanne University Hospital and University of Lausanne, Lausanne, Switzerland
15. Genomics Division, Instituto Tecnológico y de Energías Renovables (ITER), Santa Cruz de Tenerife, Spain; Research Unit, Hospital Universitario N.S. de Candelaria, Santa Cruz de Tenerife, Spain; CIBER de Enfermedades Respiratorias, Instituto de Salud Carlos III, Madrid, Spain
16. Departments of Medical Genetics & Histology and Embryology, Faculty of Medicine; Department of Translational Medicine, Health Sciences Institude, Bursa Uludağ University, Bursa, Turkey
17. Department of Paediatric Immunology and Pulmonology, Centre for Primary Immunodeficiency Ghent (CPIG), PID Research Laboratory, Jeffrey Modell Diagnosis and Research Centre, Ghent University Hospital, Ghent, Belgium
18. Sharjah Institute of Medical Research, College of Medicine, University of Sharjah, Sharjah, United Arab Emirates

19. Departments of Pediatrics, Immunology and Microbiology, University of Colorado, School of Medicine, Aurora, Colorado, USA
20. Institute for Personalized Medicine, Icahn School of Medicine at Mount Sinai, New York, NY, USA; Department of Genetics and Genomic Sciences, Icahn School of Medicine at Mount Sinai, New York, NY, USA
21. Department of Clinical Immunology and Infectious Diseases, National Research Institute of Tuberculosis and Lung Diseases, The Clinical Tuberculosis and Epidemiology Research Center, National Research Institute of Tuberculosis and Lung Diseases (NRITLD), Masih Daneshvari Hospital, Shahid Beheshti, University of Medical Sciences, Tehran, Iran
22. Hôpital Bichat, Paris, France.
23. Department of Biomedicine, Aarhus University, Aarhus, Denmark
24. A*STAR Infectious Disease Labs, Agency for Science, Technology and Research, Singapore; Lee Kong Chian School of Medicine, Nanyang Technology University, Singapore
25. Department of Biomedicine and Prevention, Tor Vergata University of Rome, Rome, Italy
26. Department of Pediatrics, Graduate School of Biomedical and Health Sciences, Hiroshima University, Hiroshima, Japan
27. Tokyo Medical and Dental University, Tokyo, Japan), David S. Perlin (Center for Discovery and Innovation, Hackensack Meridian Health, Nutley, NJ, USA
28. Laboratory of Immunogenetics of Human Diseases, Innate Immunity Group, IdiPAZ Institute for Health Research, La Paz Hospital, Madrid, Spain
29. Institut de Biomedicina de València-CSIC, CIBERNED, Unitat Mixta de Neurologia i Genètica, IIS La Fe, Vallencia, Spain
30. Center for Discovery and Innovation, Hackensack Meridian Health, Nutley, NJ, USA
31. Allergology and Clinical Immunology, Department of Medicine, Groote Schuur Hospital, University of Cape Town Lung Institute, South Africa
32. Facultad de Medicina Universidad del Desarrollo-Clínica Alemana, Unidad de Inmunología y Reumatología, Hospital Roberto del Río, Santiago, Chile Santiago, Chile
33. Faculdades Pequeno Príncipe, Instituto de Pesquisa Pelé Pequeno Príncipe, Curitiba, Brazil
34. Neurometabolic Diseases Laboratory, Bellvitge Biomedical Research Institute (IDIBELL), L'Hospitalet de Llobregat, Barcelona, Spain; Catalan Institution of Research and Advanced Studies (ICREA), Barcelona, Spain; Center for Biomedical Research on Rare Diseases (CIBERER), ISCIII, Barcelona, Spain
35. Human Evolutionary Genetics Unit, CNRS U2000, Institut Pasteur, Paris, France; Human Genomics and Evolution, Collège de France, Paris, France
36. Department of Immunology, University Hospital of Gran Canaria Dr. Negrín, Canarian Health System, Las Palmas de Gran Canaria, Spain; Department of Clinical Sciences, University Fernando Pessoa Canarias, Las Palmas de Gran Canaria, Spain
37. Department of Immunology, Second faculty of Medicine, Charles University, University Hospital Motol, Prague, Czech Republic
38. Specialized Immunology Laboratory of Dr. Shahrooei, Ahvaz, Iran; Department of Microbiology and Immunology, Clinical and Diagnostic Immunology, KU Leuven, Leuven, Belgium.
39. Central European Institute of Technology & Department of Biology, Faculty of Medicine, Masaryk University, Brno, Czech Republic.
40. Department of Medical Microbiology, University Medical Center Utrecht, Utrecht, Netherlands
41. Department of Internal Medicine II, Medical University of Innsbruck, Innsbruck, Austria
42. Garvan Institute of Medical Research, Darlinghurst, NSW, Australia; St Vincent's Clinical School, Faculty of Medicine, UNSW Sydney, NSW, Australia
43. Precision Medicine Unit, Lausanne University Hospital and University of Lausanne, Lausanne, Switzerland
44. BC Children's Hospital, The University of British Columbia, Vancouver, Canada

45. Centre for Precision Therapeutics, Genetics & Genomic Medicine Centre, Holy Family Red Crescent Medical College Dhaka, Bangladesh
46. Laboratory of Medical Genetics, IRCCS Bambino Gesù Children's Hospital, Rome, Italy
47. Hospices Civils de Lyon, Lyon, France; International Center of Research in Infectiology, Lyon University, INSERM U1111, CNRS UMR 5308, ENS, UCBL, Lyon, France.
48. San Raffaele Telethon Institute for Gene Therapy, IRCCS Ospedale San Raffaele, and Vita Salute San Raffaele University, Milan, Italy.
49. Pediatric Infectious Diseases and Immunodeficiencies Unit, Val d'Hebron, Barcelona Hospital Campus, Barcelona, Catalunya, Spain
50. Instituto Nacional de Pediatría, Mexico City, Mexico
51. Department of Medicine, Center for Hematology and Regenerative Medicine, Karolinska Institutet, Stockholm, Sweden, and Broegelmann Research Laboratory, Department of Clinical Sciences, University of Bergen, Bergen, Norway
52. Division of Experimental Medicine, McGill University Health Centre (MUHC), Montreal, Canada
53. Laboratory for Clinical Infectious and Inflammatory Disorders, Department of Microbiology, Immunology and Transplantation, KU Leuven, Leuven, Belgium
54. Department of Molecular Biology and Genetics, Bilkent University, Ankara, Turkey
55. Stem Cell Biology and Transplantation, Medical University of Varna, Bulgaria
